# Lensoside Aβ as an Adjuvant to the Anti-Glioma Potential of Sorafenib

**DOI:** 10.3390/cancers13112637

**Published:** 2021-05-27

**Authors:** Aleksandra Maciejczyk, Justyna Kapral-Piotrowska, Joanna Sumorek-Wiadro, Adrian Zając, Ewa Grela, Rafał Luchowski, Wiesław I. Gruszecki, Marta Kinga Lemieszek, Iwona Wertel, Łukasz Pecio, Jerzy Żuchowski, Krystyna Skalicka-Woźniak, Bożena Pawlikowska-Pawlęga, Monika Hułas-Stasiak, Wojciech Rzeski, Radosław Rola, Joanna Jakubowicz-Gil

**Affiliations:** 1 Department of Functional Anatomy and Cytobiology, Institute of Biological Sciences, Maria Curie-Sklodowska University, Akademicka 19, 20-033 Lublin, Poland; olamaciejczyk77@gmail.com (A.M.); justyna.kapral-piotrowska@poczta.umcs.lublin.pl (J.K.-P.); jsumorek@poczta.umcs.lublin.pl (J.S.-W.); adrian.zajac@poczta.umcs.lublin.pl (A.Z.); bozena.pawlikowska-pawlega@poczta.umcs.lublin.pl (B.P.-P.); monika.hulas-stasiak@poczta.umcs.lublin.pl (M.H.-S.); rzeskiw@hektor.umcs.lublin.pl (W.R.); 2 Independent Laboratory of Cancer Diagnostics and Immunology, 1st Chair and Department of Oncological Gynaecology and Gynaecology, Medical University of Lublin, Staszica 16, 20-081 Lublin, Poland; iwonawertel@umlub.pl; 3 Department of Biophysics, Maria Curie-Sklodowska University, Pl. M. Curie-Skłodowskiej 1, 20-031 Lublin, Poland; ewa.grela@poczta.umcs.lublin.pl (E.G.); rafal.luchowski@poczta.umcs.lublin.pl (R.L.); wieslaw.gruszecki@poczta.umcs.lublin.pl (W.I.G.); 4 Department of Medical Biology, Institute of Rural Health, Jaczewskiego 2, 20-950 Lublin, Poland; lemieszek.marta@imw.lublin.pl; 5 Department of Biochemistry and Crop Quality Institute of Soil Science and Plant Cultivation, State Research Institute, Czartoryskich 8, 24-100 Puławy, Poland; lpecio@iung.pulawy.pl (Ł.P.); jzuchowski@iung.pulawy.pl (J.Ż.); 6 Independent Laboratory of Natural Products Chemistry, Medical University of Lublin, Chodzki 1, 20-093 Lublin, Poland; kskalicka@pharmacognosy.org; 7 Department of Neurosurgery and Pediatric Neurosurgery, Medical University of Lublin, Jaczewskiego 8, 20-954 Lublin, Poland; radoslaw.rola@umlub.pl

**Keywords:** lensoside Aβ, sorafenib, gliomas, apoptosis, FTIR, FLIM, SEM

## Abstract

**Simple Summary:**

Flavonoids are plant polyphenolic secondary metabolites, commonly consumed in the human diet. Lensoside Aβ is a quercetin glycoside isolated from the aerial parts of lentil (*Lens culinaris*) organs. The activity of this secondary metabolite, especially in terms of its anticancer potential, has been poorly studied. Currently, there are no published data about the effect of this flavonoid on gliomas, which are so-far incurable, aggressive neoplasms of the central nervous system with a highly infiltrative character. In this study, we found that lensoside Aβ itself exhibits poor anti-glioma properties but exerts a strongly potentiated effect in combination with sorafenib (inhibitor of Raf kinase) on apoptosis induction in cancer cells. Our results have shown that sorafenib with lensoside Aβ seems to be a promising combination that might be useful in glioma therapy. Additionally, the former observation, pointing to the key role of flavonoids as adjuvants in chemotherapy, is confirmed.

**Abstract:**

Aim: The anti-glioma effect of lensoside Aβ alone and in combination with sorafenib (pro-survival Raf kinase inhibitor) was evaluated for the first time in terms of programmed cell death induction in anaplastic astrocytoma and glioblastoma multiforme cell lines as an experimental model. Apoptosis, autophagy, and necrosis were identified microscopically (fluorescence and scanning microscopes) and confirmed by flow cytometry (mitochondrial membrane potential MMP and cell death). The expression of apoptotic (caspase 3) and autophagic markers (beclin 1) as well as Raf kinase were estimated by immunoblotting. The FTIR method was used to determine the interaction of the studied drugs with lipid and protein groups within cells, while the modes of drug action within the cells were assessed with the FLIM technique. Results: Lensoside Aβ itself does not exhibit anti-glioma activity but significantly enhances the anti-cancer potential of sorafenib, initiating mainly apoptosis of up to 90% of cells. It was correlated with an increased level of active caspase 3, a reduced MMP value, and a lower level of Raf kinase. The interaction with membrane structures led to morphological changes typical of programmed death. Conclusions: Our results indicate that lensoside Aβ plays an important role as an adjuvant in chemotherapy with sorafenib and may be a potential candidate in anti-glioma combination therapy.

## 1. Introduction

Legumes are the oldest crops cultivated by humans and one of the most important foodstuffs consumed in the daily diet [1]. They are a significant source of proteins, dietary fibers, carbohydrates, and minerals [2]. Among legumes, lentils (*Lens culinaris*) have been gaining popularity for their nutritive value due to the high content of biological and active compounds. They are an excellent source of carbohydrates (especially raffinose family oligosaccharides, RFOs), proteins, and vitamins [3,4]. While there is rich literature on the basic nutrients of lentil grain, the number of publications about lentil secondary metabolites is more limited. The seeds of this plant are known to contain phytosterols, phytic acid, saponins, and phenolic compounds. The last group is represented by condensed tannins (present in significant amounts in the seed coat), phenolic acids, lignans, stilbenes, and flavonoids. Flavonoids, which are widely distributed in the plant kingdom, deserve special attention [5,6,7]. These common food constituents are extensively investigated due to their antioxidant properties, diverse biological activities, and role in prevention of cardiovascular diseases and cancer [8,9]. Lentil flavonoids comprise mainly catechin and glycosidic derivatives of kaempferol, quercetin, myricetin, apigenin, and luteolin, isolated mainly from seeds [10,11,12]. Very little is known about the flavonoids present in leaves and stems [13,14,15]. Żuchowski et al. [16,17] showed that the aerial parts of lentil are a rich source of natural polyphenolic compounds, especially kaempferol and quercetin derivatives. One of them is lensoside Aβ (LAβ), representing quercetin glycoside. As presented in the first (and the only so far) article, it is able to intercalate into the cell membrane and interact with its structures, which in consequence may have beneficial effects [18]. Our previous experiments revealed that quercetin has anticancer properties, with special emphasis on glioma cells [19,20]. Gliomas are the most common and most malignant primary tumors of the central nervous system. They are characterized by aggressive proliferation and growth as well as diffusive infiltration into adjacent brain parenchyma. They are also very resistant to elimination via programmed death upon chemotherapy [21,22,23]. Such a phenomenon may be correlated with the presence of mutations within genes, the products of which are involved in the pro-survival pathways responsible for the regulation of cell proliferation and differentiation. An example of such a mechanism is the amplification of the Ras–Raf–MEK–ERK (Ras—Ras protein; Raf—rapidly accelerated fibrosarcoma kinase; MEK—mitogen-activated protein kinase; ERK—extracellular signal-regulated kinase) pathway in glioma cells [24]. This signal transmission may be blocked by sorafenib, i.e., a targeted cancer drug used in advanced renal cell carcinoma (RCC), unresectable hepatocellular carcinomas (HCC), and thyroid cancer, showing special affinity to Raf kinase. The interest in this drug was aroused by the information about possible anti-glioma activity. It was shown to inhibit new blood vessel formation and cell proliferation, which led to initiation of autophagy or apoptosis [25,26]. As shown in previous research, the anti-glioma potential of sorafenib was enhanced by quercetin [27]. Therefore, encouraged by the results and promising properties of the quercetin derivative lensoside Aβ, the present pioneer study was carried out to evaluate for the first time the anti-glioma effect of lensoside Aβ alone and in combination with sorafenib (Raf kinase inhibitor) in terms of programmed cell death induction. The experiments were conducted on anaplastic astrocytoma (AA, WHO grade III, lines MOGGCCM, LN229, and SW1783) and glioblastoma multiforme (GBM, WHO grade IV, lines T98G and LN-18) cells, belonging to the group of the most malignant tumors of the central nervous system.

## 2. Results

### 2.1. Sensitivity of Malignant Glioma Cell Lines to Lensoside Aβ and Sorafenib Application

**Figure 1:**
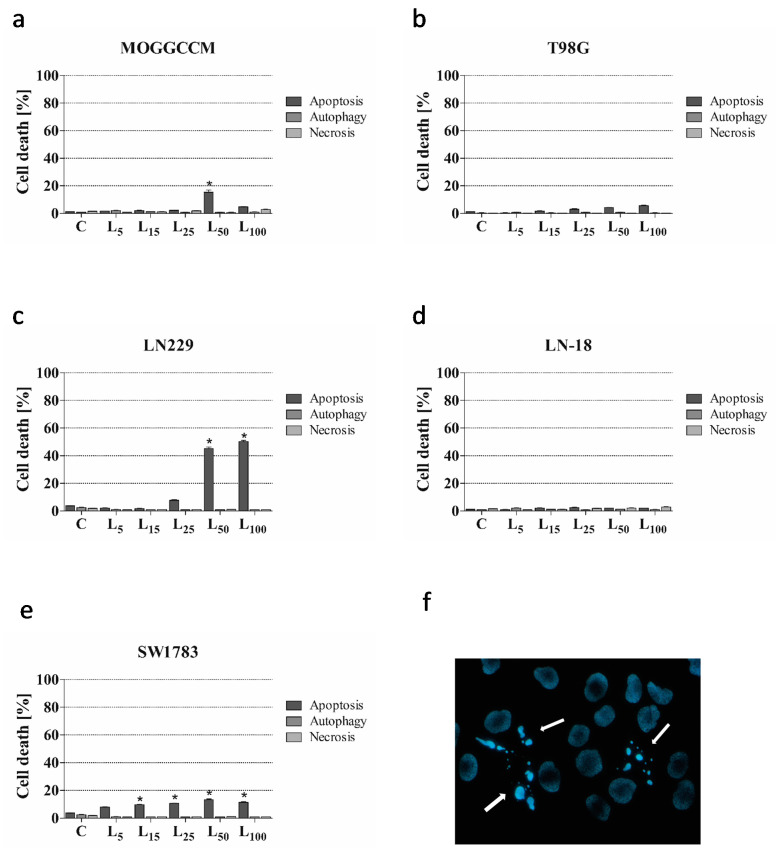
Effect of different concentrations of lensoside Aβ on the level of apoptosis, autophagy, and necrosis, identified microscopically after staining with Hoechst 33342, acridine orange, and propidium iodide, respectively, in anaplastic astrocytoma (MOGGCCM, LN229, SW1783; (**a**,**c**,**e**)) and glioblastoma multiforme cells (T98G, LN-18; (**b**,**d**)) incubated with the compound for 24 h. (**f**) Representative picture of apoptotic MOGGCCM cells (pointed by arrows) stained with Hoechst 33342 after 50 µM lensoside Aβ treatment; C—control; * *p* < 0.01 compared to the control.

**Figure 2:**
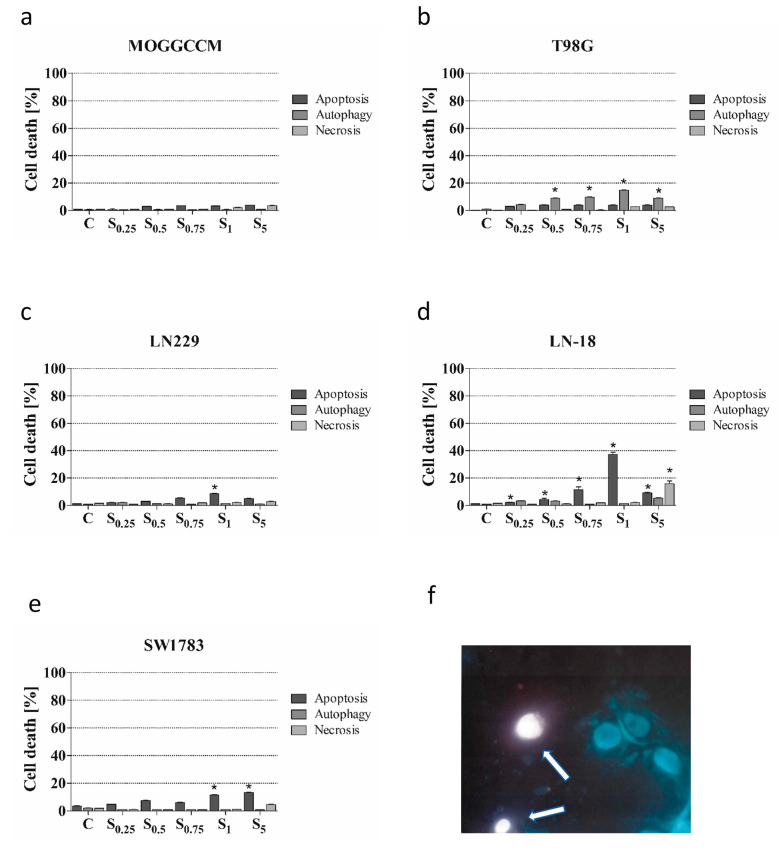
Effect of sorafenib on the level of apoptosis, autophagy, and necrosis, identified microscopically after staining with Hoechst 33342, acridine orange, and propidium iodide, respectively, in anaplastic astrocytoma (MOGGCCM, LN229, SW1783; (**a**,**c**,**e**)) and glioblastoma multiforme cells (T98G, LN-18; (**b**,**d**,**f**)) incubated with the compound for 24 h. (**f**) Representative picture of necrotic LN-18 cells (pointed by arrows) stained with propidium iodide after 5 µM sorafenib treatment; C—control; * *p* < 0.01 compared to the control.

**Figure 3:**
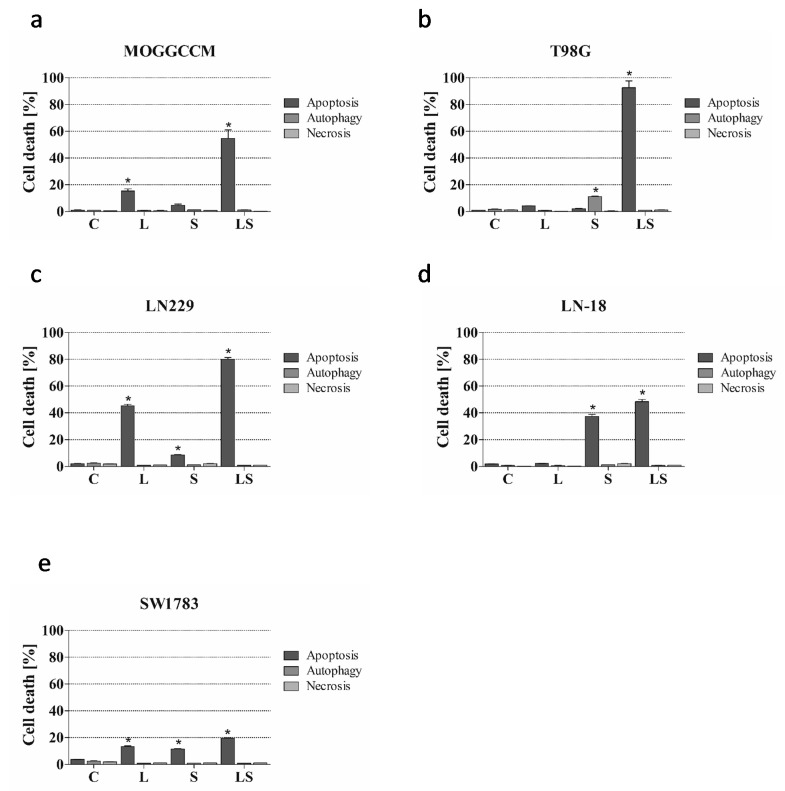
Effect of lensoside Aβ (L) and sorafenib (S) applied alone and in combination (LS) on the level of apoptosis, autophagy, and necrosis, identified microscopically after staining with Hoechst 33342, acridine orange, and propidium iodide, respectively, in anaplastic astrocytoma (MOGGCCM, LN229, SW1783; (**a**,**c**,**e**)) and glioblastoma multiforme cells (T98G, LN-18; (**b**,**d**)) incubated with the compound for 24 h. C—control; * *p* < 0.01 compared to the control.

To estimate the sensitivity of glioma cells to the lensoside Aβ and/or sorafenib treatment, a staining method with dyes specific for apoptosis, necrosis, and autophagy, namely, Hoechst 33342, propidium iodide, and acridine orange, respectively, was employed (Figure 1, Figure 2 and Figure 3).

#### 2.1.1. Lensoside Aβ

The microscopic observation revealed that lensoside Aβ applied at the concentration of 5–100 µM to the glioblastoma multiforme LN-18 and T98G culture medium for 24 h had no significant effect on cell death (Figure 1b,d). A pro-apoptotic effect was observed in the anaplastic astrocytoma cells (MOGGCCM, LN229, SW1783) (Figure 1a,c,e). In the case of LN229, lensoside Aβ initiated apoptosis in 45.3% and 50.3% of cells at the concentration of 50 and 100 µM, respectively. A weaker but still significant pro-apoptotic effect was observed in the MOGGCCM (15.3%) cell line after incubation with the flavonoid at the concentration of 50 µM. A decrease in the number of apoptotic cells was observed in the MOGGCCM line after treatment with lensoside Aβ 100 µM. It may be correlated with the increased level of necrosis within a cell population (but not significant) as well as increased detachment of already dead cells (less culture dense compared to the control). Such a reaction may also be related to the specificity of the cell line while a very similar reaction to the flavonoid quercetin was observed and such an effect was associated with a necrotic state [20]. Line SW1783 also appeared to be less sensitive to the action of lensoside Aβ, which initiated apoptosis with a maximum level of 13.3% at the concentration of 50 µM. Lensoside Aβ had no significant effect on the induction of necrosis and autophagy in the examined cell lines. Based on the results obtained, lensoside Aβ at a concentration of 50 µM in the case of MOGGCCM, T98G, LN229, and SW1783 was chosen for further experiments. In the case of LN-18, showing no sensitivity to the induction of apoptosis upon the flavonoid treatment, a concentration 25 µM was selected, according to the Chou-Talalay analysis (Figure A4). Lensoside Aβ did not initiate any type of death in normal oligodendrocytes and the level of apoptosis necrosis or autophagy was comparable to the control (Figure S2).

#### 2.1.2. Sorafenib

Our previous study showed that sorafenib applied at the concentration of 0–5 µM to the MOGGCCM culture medium had no significant effect on cell death, while autophagy was dominant in the T98G line [27] (Figure 2a,b). Interestingly, sorafenib was the most effective inducer of apoptosis in the LN-18 cell line (Figure 2d). This drug at the concentration of 1 µM initiated apoptosis in 39% of the cells. The other concentrations used (0.25, 0.5, 0.75, and 5 µM) also increased the number of apoptotic cells, but not as spectacularly. This is especially interesting in the case of a high dose of sorafenib (5 µM), where the level of apoptosis compared to the concentration of 1 µM was lower but accompanied by a significant increase in the number of necrotic cells (18%) (Figure 2d,f). It means that this concentration initiates injury rather than a programmed type of death and therefore it is not recommended to use such a dose in treatment. The LN229 and SW1783 lines appeared to be less sensitive to sorafenib (Figure 2c,e). It induced apoptosis with a maximum level of 8.6% and 11.5% at the concentration of 1 µM, respectively. Furthermore, in the SW1783 cell line, sorafenib at the concentration of 5 µM induced apoptosis in 13.2% of the cells, but it was accompanied by necrosis in approximately 5% of the cells (Figure 2e). Additionally, sorafenib did not initiate any type of death in normal cells and the level of apoptosis necrosis or autophagy was comparable to the control (Figure S2).

#### 2.1.3. Combination of Lensoside Aβ and Sorafenib

**Figure A1:**
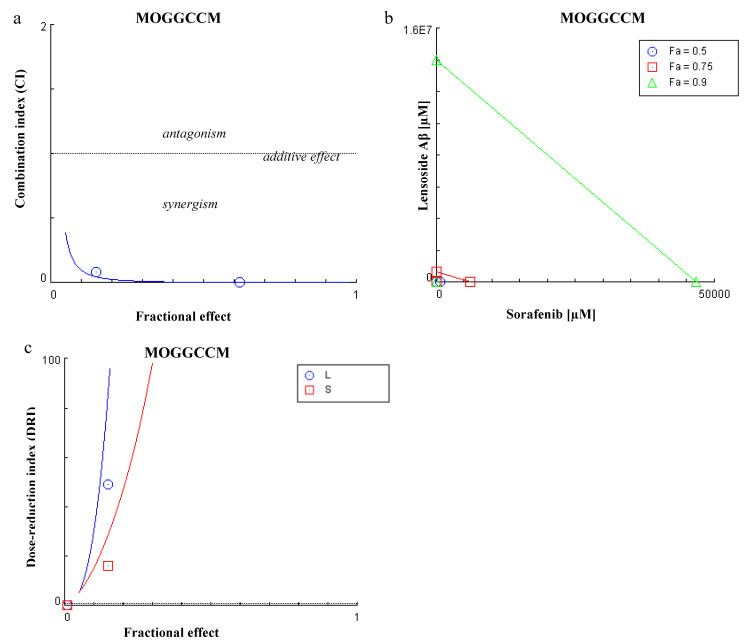
Lensoside Aβ (L) and sorafenib (S) combination treatment. (**a**) Combination index (CI) plot: The combination index is plotted as a function of Fa (fractional effect). (**b**) Isobologram for combination: isobologram at IC50, IC75, and IC90. (**c**) the Fa-DRI (dose reduction index) plot (Chou-Martin plot). Combination index (CI) and dose reduction index (DRI) were calculated according to the method of Chou and Talalay using the Compusyn software. CI < 1, CI = 1 and CI > 1 indicate synergistic effect, additive effect, and antagonistic effect, respectively. The combination of lensoside Aβ with sorafenib in the MOGGCCM line showed a synergistic effect. It was stronger when the higher dose was used, which is extremely important in anti-cancer therapy (for IC97, CI = 0.0001). Moreover, the combination treatment significantly reduced the doses of both drugs (DRI > 1), which in a single application would have to be higher to obtain the same effect. The final concentration of lensoside Aβ and sorafenib used in further studies was 50 µM and 1 µM, respectively.

**Figure A2:**
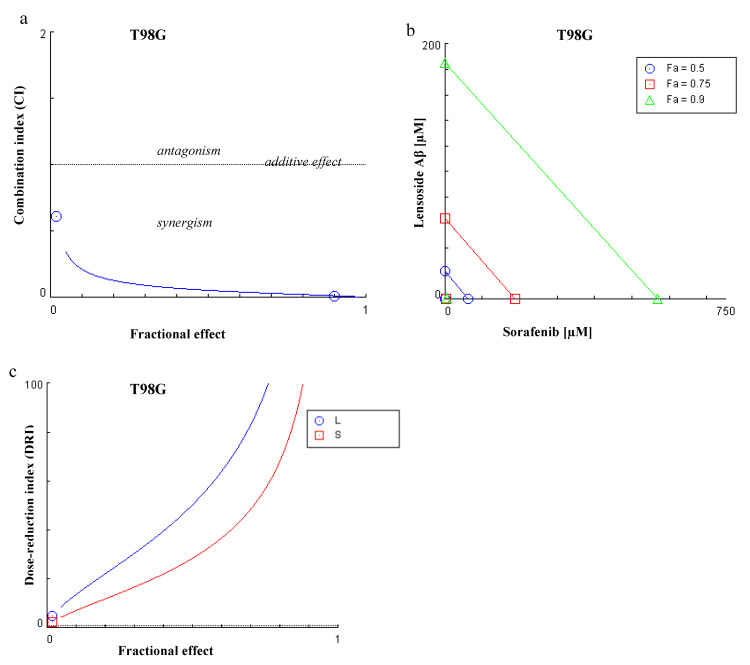
Lensoside Aβ (L) and sorafenib (S) combination treatment. (**a**) Combination index (CI) plot: The combination index is plotted as a function of Fa (fractional effect). (**b**) Isobologram for combination: isobologram at IC50, IC75, and IC90. (**c**) the Fa-DRI (dose reduction index) plot (Chou-Martin plot). Combination index (CI) and dose reduction index (DRI) were calculated according to the method of Chou and Talalay using the Compusyn software. CI < 1, CI = 1 and CI > 1 indicate synergistic effect, additive effect, and antagonistic effect, respectively. The combination of lensoside Aβ with sorafenib in the T98G line showed a synergistic effect. It was stronger when the higher dose was used, which is extremely important in anti-cancer therapy (for IC97, CI = 0.01). Moreover, the combination treatment significantly reduced the doses of both drugs (DRI > 1), which in a single application would have to be higher to obtain the same effect. For the experimentally determined dose (50 µM of lensoside Aβ and 1 µM of sorafenib), the CI and Fa were 0.01 and 0.9, respectively. Therefore, these concentrations were used in the research.

**Figure A3:**
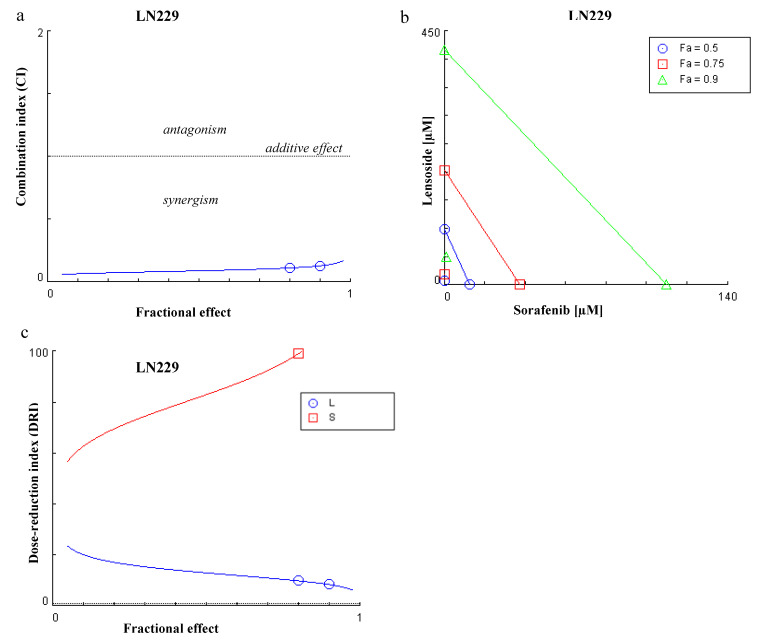
Lensoside Aβ (L) and sorafenib (S) combination treatment. (**a**) Combination index (CI) plot: The combination index is plotted as a function of Fa (fractional effect). (**b**) Isobologram for combination: isobologram at IC50, IC75, and IC90. (**c**) the F_a_-DRI (dose reduction index) plot (Chou-Martin plot). Combination index (CI) and dose reduction index (DRI) were calculated according to the method of Chou and Talalay using the Compusyn software. CI < 1, CI = 1 and CI > 1 indicate synergistic effect, additive effect, and antagonistic effect, respectively. The combination of lensoside Aβ with sorafenib in the LN229 line showed a synergistic effect. For the experimentally determined dose (1 µM of sorafenib and 50 µM of lensoside Aβ), the CI and Fa were 0.12 and 0.9 respectively. That means, the concentrations of used compounds, induced programmed death in 90% of cells.

**Figure A4:**
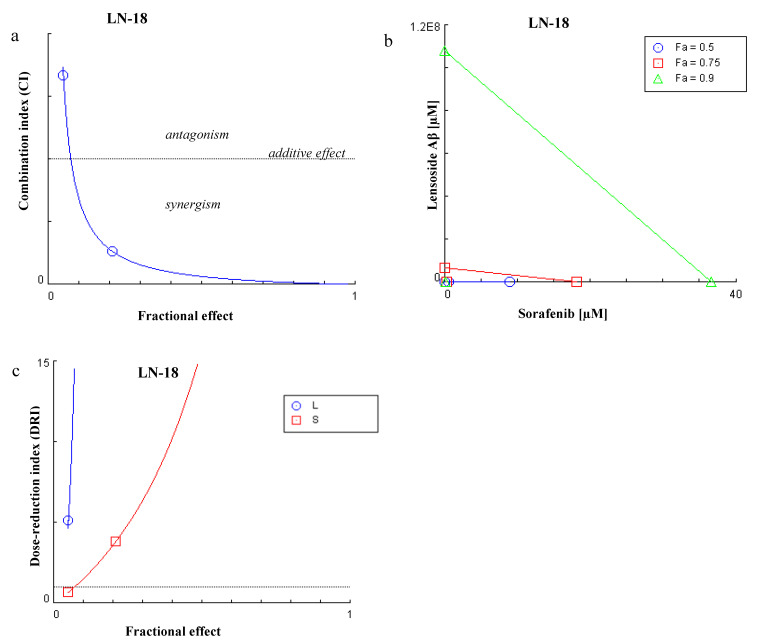
Lensoside Aβ (L) and sorafenib (S) combination treatment. (**a**) Combination index (CI) plot: The combination index is plotted as a function of Fa (fractional effect). (**b**) Isobologram for combination: isobologram at IC50, IC75, and IC90. (**c**) the F_a_-DRI (dose reduction index) plot (Chou-Martin plot). Combination index (CI) and dose reduction index (DRI) were calculated according to the method of Chou and Talalay using the Compusyn software. CI < 1, CI = 1 and CI > 1 indicate synergistic effect, additive effect, and antagonistic effect, respectively. Synergy (CI < 1) at high effect levels (at fa > 0.90) is more relevant to anticancer therapeutic effect than the CI at low effect levels (at fa < 0.3). The combination of lensoside Aβ with sorafenib in the LN-18 line showed a synergistic effect at fa > 0.1. In the present case in isobologram, combination data point (1 µM sorafenib and 25 µM lensoside Aβ) on the lower left indicates synergism at all IC.

**Figure A5:**
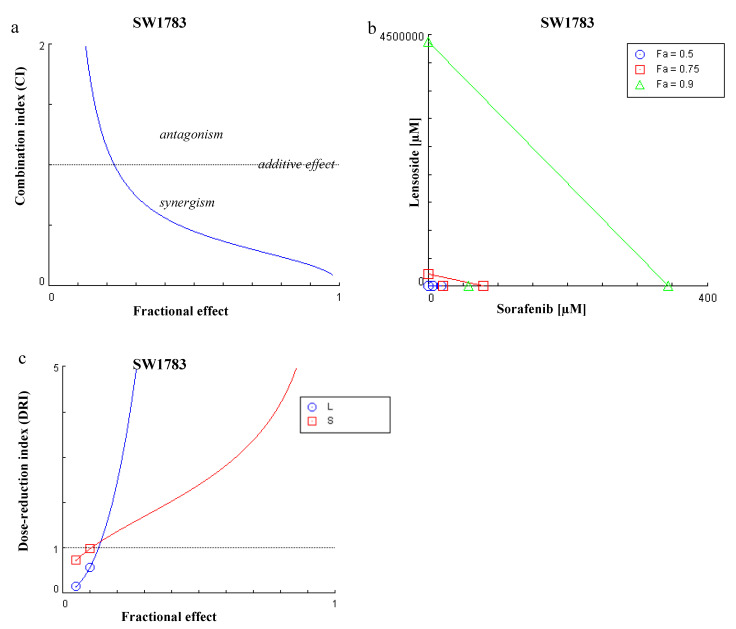
Lensoside Aβ (L) and sorafenib (S) combination treatment. (**a**) Combination index (CI) plot: The combination index is plotted as a function of Fa (fractional effect). (**b**) Isobologram for combination: isobologram at IC50, IC75, and IC90. (**c**) the F_a_-DRI (dose reduction index) plot (Chou-Martin plot). Combination index (CI) and dose reduction index (DRI) were calculated according to the method of Chou and Talalay using the Compusyn software. CI < 1, CI = 1 and CI > 1 indicate synergistic effect, additive effect, and antagonistic effect, respectively. The combination of lensoside Aβ with sorafenib in the SW1783 line showed a synergistic effect. It was stronger when the higher dose was used, which is extremely important in anti-cancer therapy (for IC97, CI = 0.1). Moreover, the combination treatment significantly reduced the doses of both drugs (DRI > 1), which in a single application would have to be higher to obtain the same effect. Therefore, in the combined therapy of lensoside Aβ with sorafenib, the lowest doses that gave the best effect were used. These were the same as for monotherapy and the final concentrations of sorafenib and lensoside Aβ were 1 µM and 50 µM, respectively.

Based on the results described previously and the Chou–Talalay method (Appendix A, Figure A1, Figure A2, Figure A3, Figure A4 and Figure A5), the optimal concentration of sorafenib (1 µM) and lensoside Aβ (50 µM for MOGGCCM, T98G, LN229, and SW1783 and 25 µM for LN-18) was selected to examine the combined effect of lensoside Aβ with sorafenib, which was confirmed experimentally 

Interestingly, the combined treatment of all five cell lines with lensoside Aβ and sorafenib was more effective in programmed cell death induction than the separate application and exerted a synergistic effect (Figure 3 and Figure A1, Figure A2, Figure A3, Figure A4 and Figure A5). Apoptosis was the dominant type of death in all experimental variants. Both drugs showed the highest pro-apoptotic activity in the T98G and LN229 cell lines, causing this type of death in 92.7% and 88.5% of the cell populations, respectively (Figure 3b,c). The percentage of apoptotic cells reached 54.7 (Figure 3a) in the MOGGCCM cell line and 46 in LN-18 (Figure 3d). The SW1783 cell line appeared to be the least sensitive to the combined action of lensoside Aβ with sorafenib. It resulted in apoptosis in only 19.5% of the cells, which was still more effective than in the case of the single administration of the examined compounds (Figure 3e). Lensoside Aβ and sorafenib had no impact on the death induction in normal oligodendrocytes and the level of apoptosis necrosis or autophagy was similar to the control (Figure S2).

### 2.2. Effect of Lensoside Aβ and Sorafenib on Apoptosis, Necrosis, and Mitochondrial Membrane Potential Estimated by Flow Cytometry

**Figure 4:**
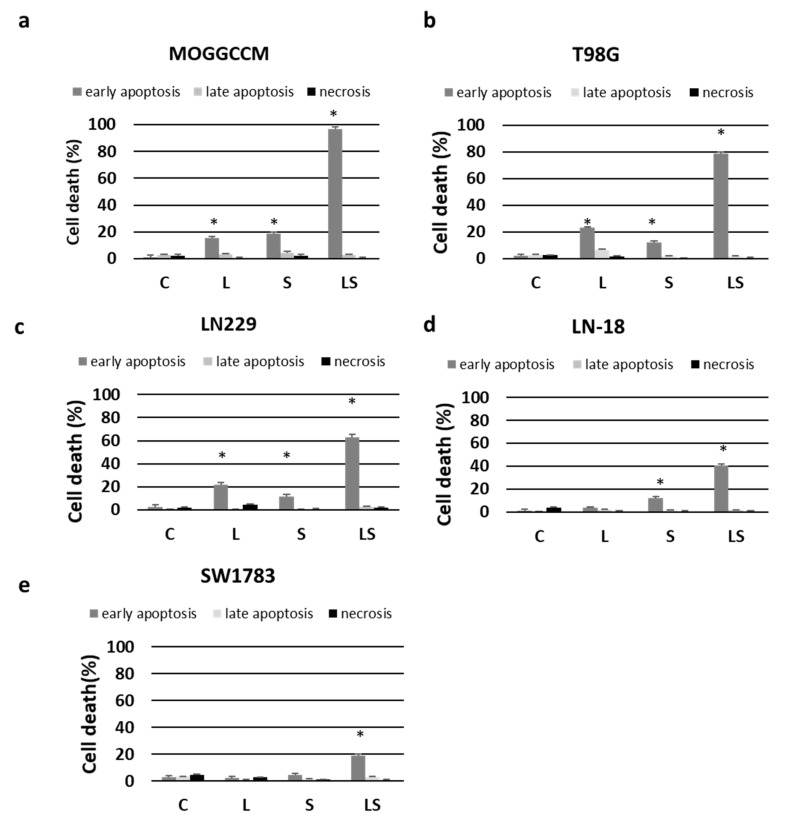
Effect of lensoside Aβ (L) and sorafenib (S) on apoptosis and necrosis induction in anaplastic astrocytoma (MOGGCCM, LN229, SW1783; (**a**,**c**,**e**)) and glioblastoma multiforme cells (T98G, LN-18; (**b**,**d**)) incubated with the compound for 24 h. Flow cytometry analysis with an Annexin V-FITC detection kit. C—control; * *p* < 0.01 compared to the control.

To confirm the reliability of the results of the microscopic observations, the glioma cell lines were treated with lensoside Aβ and sorafenib for 24 h separately and in combination of both drugs and analyzed by flow cytometry to determine the type of cell death (apoptosis or necrosis) and the mitochondrial membrane potential. The incubation of the cells with the flavonoid confirmed that LAβ induced mainly apoptosis at the early stage of the process (Figure 4). The T98G and LN229 cells appeared to be the most sensitive lines, and this type of death was observed in about 20% of the cells (Figure 4b,c). The MOGGCCM cell line turned out to be the third in terms of sensitivity, as apoptosis was observed in 16% of this cell population (Figure 4a). Cell lines SW1783 and LN-18 were resistant to the lensoside Aβ treatment, and no significant changes in the percent of apoptosis were observed in comparison with the control. Similar to lensoside Aβ, sorafenib alone induced mainly early apoptosis in all the cell lines at a level below 20% (Figure 4). The most significant effect was observed in the MOGGCCM cells, where the sorafenib treatment resulted in 19% apoptosis (Figure 4a). The most spectacular anti-glioma potential was observed when lensoside Aβ and sorafenib were added to the culture medium together. Both drugs eliminated 97% of the MOGGCCM cells and 80% of the T98G cell population via early apoptosis (Figure 4a,b). A very significant effect was also observed in the LN229 (63%) and LN-18 (41%) lines (Figure 4c,d). The SW1783 cells appeared to be the most resistant to the treatment with both drugs, but still the number of apoptotic cells reached 19% (Figure 4e).

**Figure 5:**
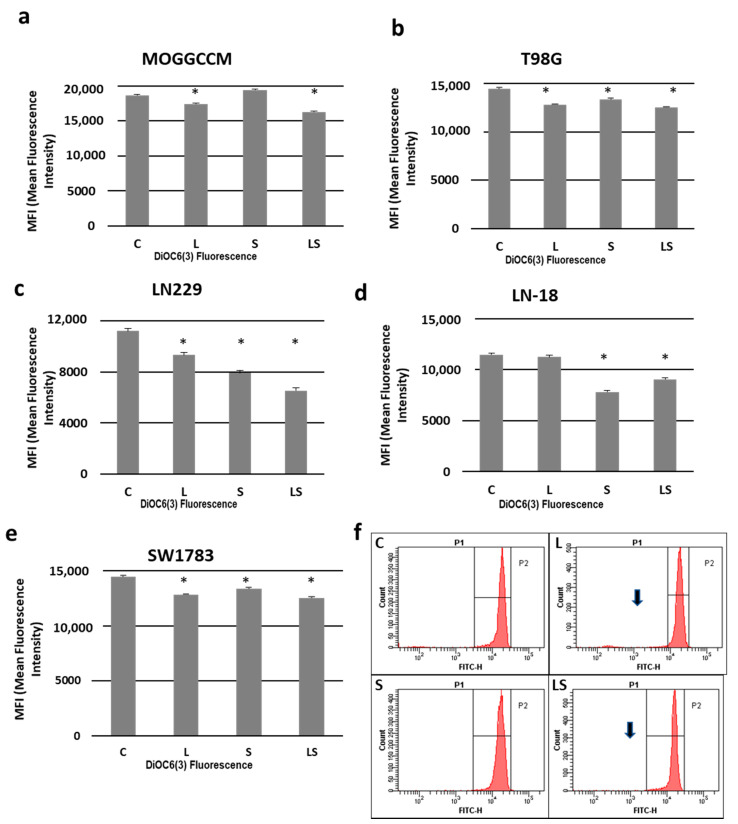
Effect of lensoside Aβ (L) and sorafenib (S) applied alone or in combination (LS) on the mitochondrial membrane potential (MMP) expressed as DiOC6(3) mean fluorescence intensity as diagrams (**a**–**e**) in anaplastic astrocytoma (MOGGCCM, LN229, SW1783; (**a**,**c**,**e**)) and glioblastoma multiforme cells (T98G, LN-18; (**b**,**d**)). (**f**) Representative gates (histograms) of the MOGGCCM cells (diminished MMP indicated by arrows). C—control; * *p* < 0.01.

A reduced mitochondrial membrane potential is one of the pro-apoptotic factors. In our experiment, lensoside Aβ and sorafenib applied alone or in combination decreased this potential in the studied cell lines (Figure 5). The lowest value was observed after the simultaneous drug treatment of the LN229 cells (Figure 5c). No significant changes in comparison with the control were observed, barring two cases: in the MOGGCCM cells incubated with sorafenib (Figure 5a) and LN-18 treated with lensoside Aβ (Figure 5d).

### 2.3. Estimation of the Level of Caspase 3, Beclin 1, and Raf Kinase upon Lensoside Aβ and Sorafenib Treatment

**Figure 6:**
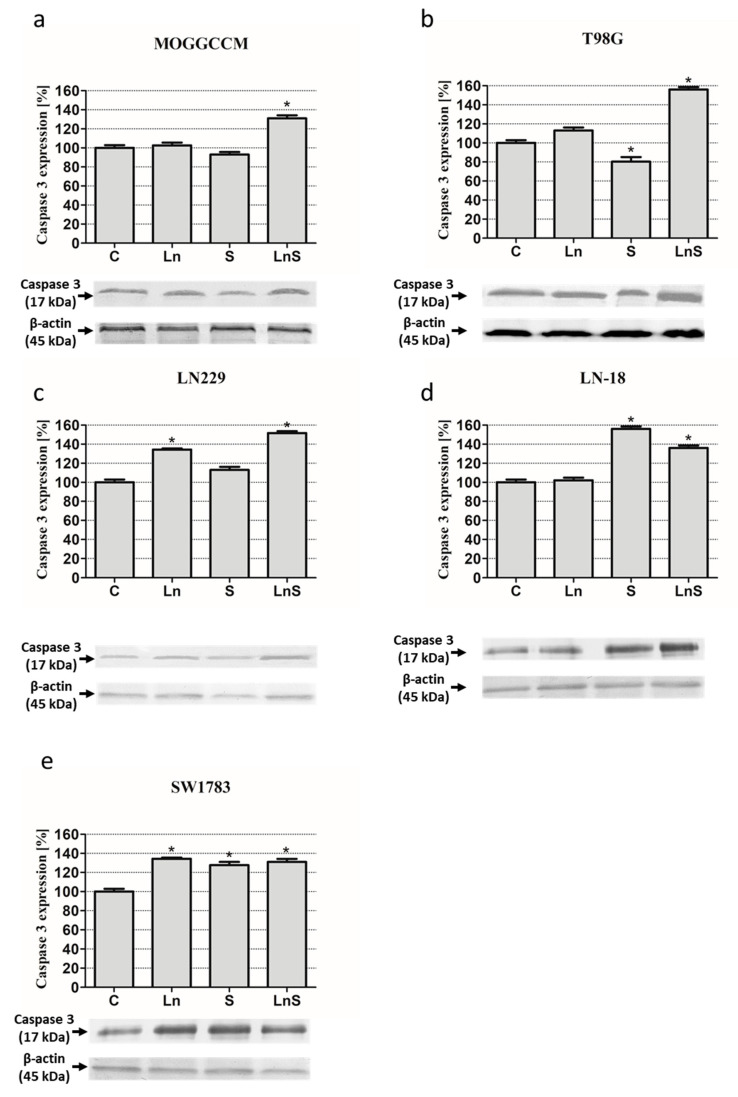
Level of active caspase 3 with representative blots in MOGGCCM (**a**), T98G (**b**), LN229 (**c**), LN-18 (**d**), and SW1783 (**e**) cells after treatment with lensoside Aβ (L) and sorafenib (S) applied alone or in combination (LS). C—control; * *p* < 0.01 compared to the control.

Programmed cell death is characterized by changes at the subcellular and molecular levels. Therefore, to confirm the reliability of the results of the microscopic observations and flow cytometry method, we decided to evaluate the effect of lensoside Aβ and sorafenib on the level of pro-apoptotic caspase 3, pro-autophagic Beclin 1, and pro-survival Raf kinase by immunoblotting. Quantitative and qualitative analyses of the immunoblots revealed that lensoside Aβ and sorafenib applied in combination significantly increased the level of active caspase 3 in all the cell lines (Figure 6). In the case of sorafenib alone, elevation of the level of the protein was observed in LN229, SW1783, and LN-18. Inhibition was found only in T98G. Lensoside Aβ was not very effective in increasing the level of caspase 3, and such an effect was observed only in LN229 and SW1783.

**Figure 7:**
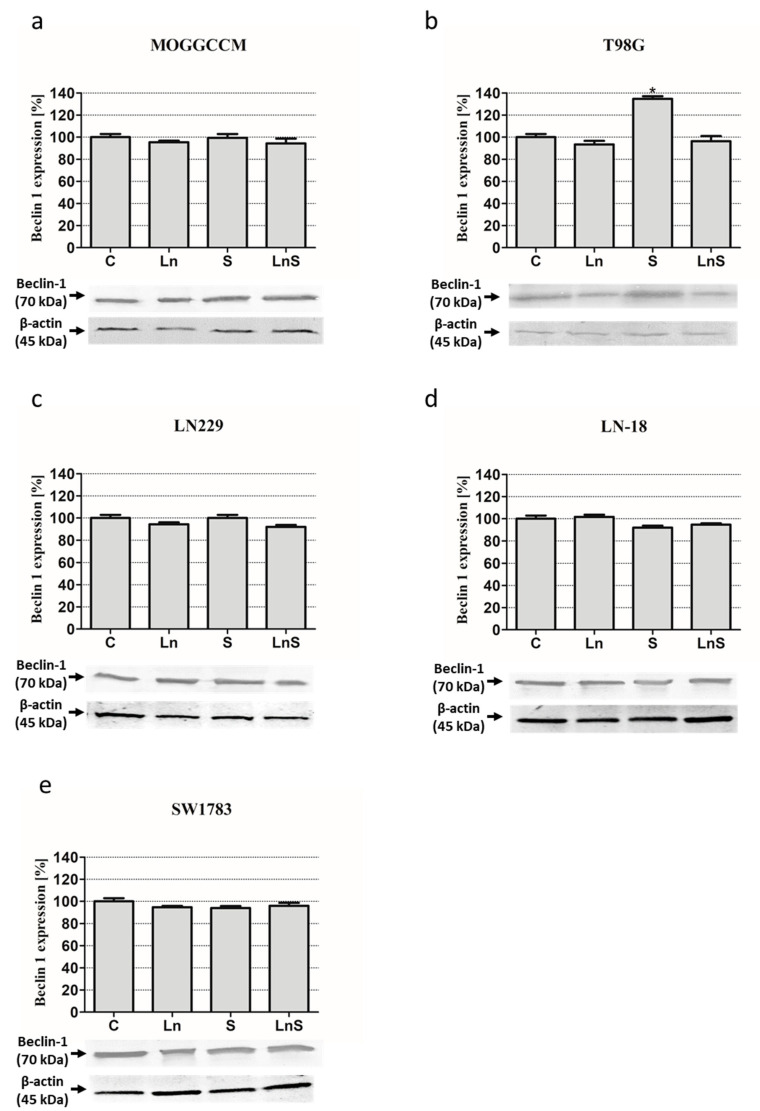
Level of beclin 1 with representative blots in MOGGCCM (**a**), T98G (**b**), LN229 (**c**), LN-18 (**d**), and SW1783 (**e**) cells after treatment with lensoside Aβ (L) and sorafenib (S) applied alone or in combination (LS). C—control; * *p* < 0.01 compared to the control.

In the case of beclin 1 (Figure 7), lensoside Aβ and sorafenib had no significant effect on the protein expression in all the cells, with one exception: in the T98G cell line after the separate sorafenib treatment (Figure 7b).

**Figure 8:**
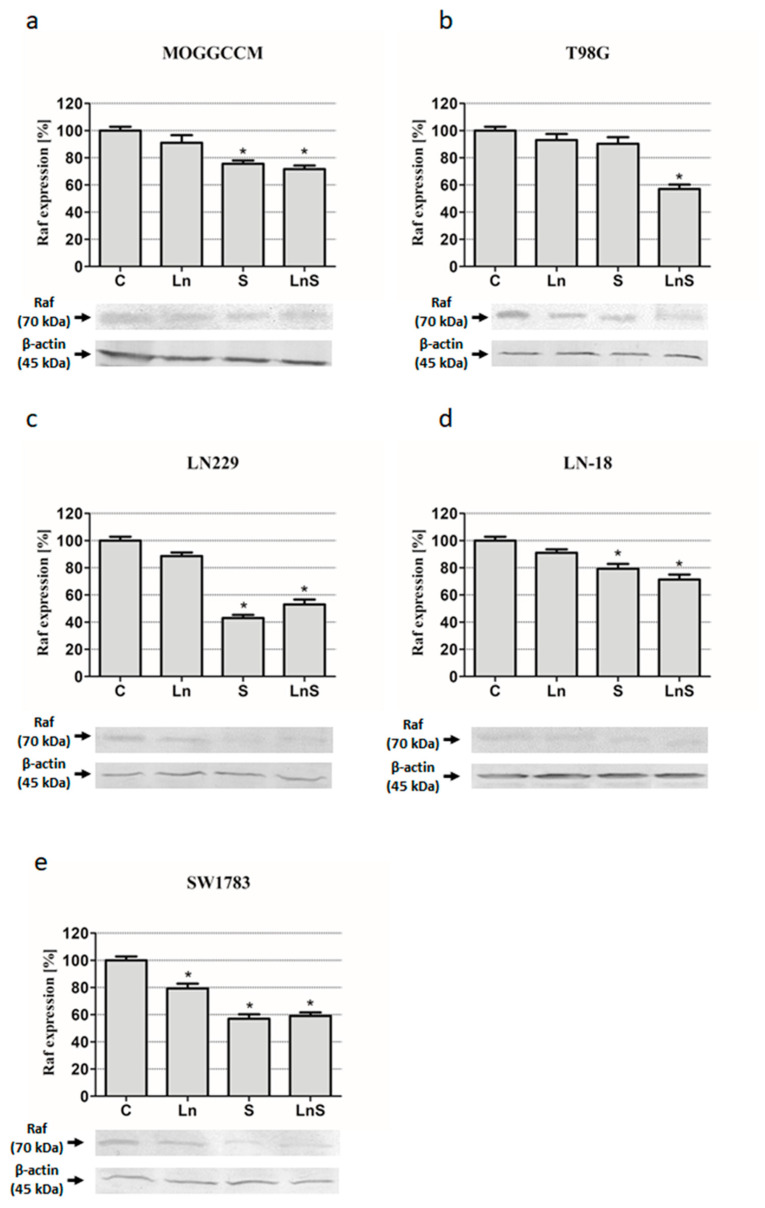
Level of Raf with representative blots in MOGGCCM (**a**), T98G (**b**), LN229 (**c**), LN-18 (**d**), and SW1783 (**e**) cells after treatment with lensoside Aβ (L) and sorafenib (S) applied alone or in combination (LS). C—control; * *p* < 0.01 compared to the control.

Sorafenib applied alone and in combination with lensoside Aβ appeared to be a very effective inhibitor of Raf kinase in MOGGCCM, LN229, LN-18, and SW1783 (Figure 8). In the last cell line, the inhibitory potential was also recorded in the case of lensoside Aβ (Figure 8e). In the T98G cells, a reduced level of Raf kinase expression was observed only after the simultaneous lensoside Aβ and sorafenib treatment (Figure 8b).

### 2.4. Scanning Electron Microscopy of Cells Treated with Lensoside Aβ and/or Sorafenib

**Figure 9:**
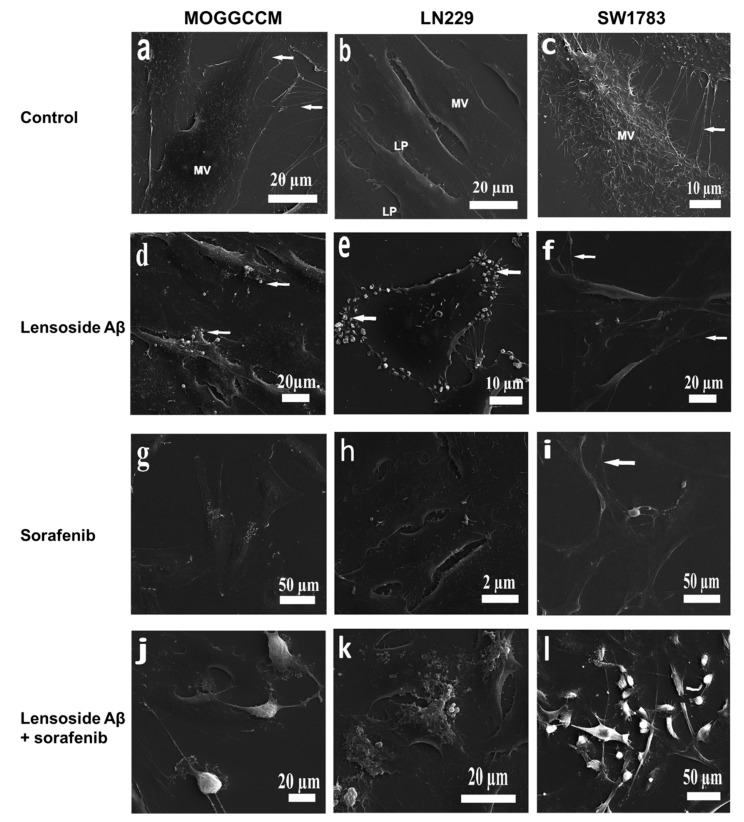
MOGGCCM, LN229, and SW1783 cells examined by scanning electron microscopy (SEM): (**a**–**c**) control cells; (**d**–**f**) lensoside Aβ-treated cells; (**g**–**i**) cells incubated with sorafenib; (**j**–**l**) combined treatment with lensoside Aβ and sorafenib. (**a**) Adherent MOGGCCM cells presenting microvilli on the surface and thin processes joining neighboring cells (arrows). (**b**) LN229 cells with scarce microvilli (MV) and lamellipodia (LP). (**c**) SW1783 cells exhibiting single shorter and longer filopodia and thin, long projections between adjacent cells (arrows). Cells are covered by dense microvilli (MV). (**d**) MOGGCCM cells after lensoside Aβ treatment showing blebs (vesicles) on the surface of the cells (arrows). (**e**) LN229 cells with altered peripheries of the cell (arrows) after lensoside Aβ treatment. (**f**) SW1783 cells connected by TNT-like processes with vesicles. (**g**) Ruffles and small vesicles on the surface of MOGGCCM cells. (**h**) LN229 cells showing microvilli on the surface of the cells. (**i**) SW1783 cells with no microvilli and long interconnections between cells (arrow). (**j**) MOGGCCM cells with apparent long TNT-like processes and LP (lamellipodia). Cells exhibit globular-shaped bodies. (**k**) LN 229 cells presenting microvesicles, blebs, and ruffles on the surface. (**l**) SW1783 cells with microvesicles on the edges and blebs formed. Some cells show a normal topography.

**Figure 10:**
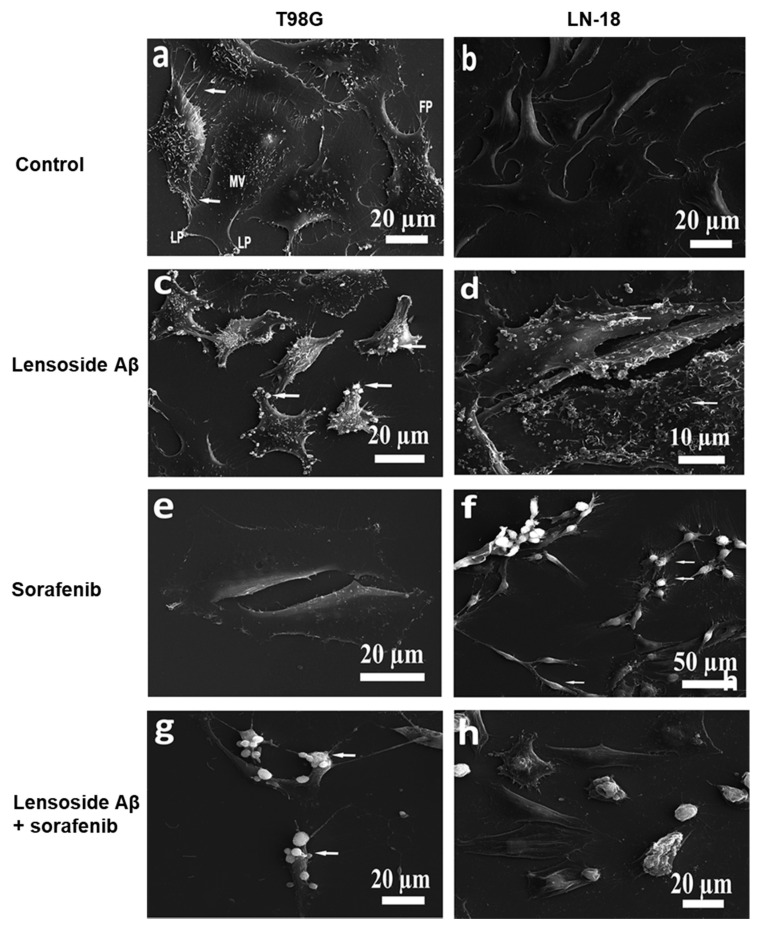
Characteristics of the surface phenotypes of the control T98G and LN-18 cells (**a**,**b**) and cells treated with lensoside Aβ (**c**,**d**), sorafenib alone (**e**,**f**), and with a combination of both drugs (**g**,**h**). (**a**) Electron micrograph of the T98G cells showing lamellipodia (LP), filopodia (FP), microvilli (MV), and long, thin, parallel projections (arrows) connecting adjacent cells. (**b**) Adherent and polygonal-shaped LN-18 cells. (**c**) Electron micrograph showing many microvesicles on the edges of T98G cells incubated with lensoside Aβ (arrows). (**d**) LN-18 cells with ruffles and blebs on the surface. (**e**) Visible loss of interconnections and microvilli in T98G cells. (**f**) LN-18 cells with prominent bundles of thin processes and deformed bodies of cells. (**g**) T98G cells with lost microvilli; blebs are visible on the surface. (**h**) LN-18 cells with ruffles on the surface, deformed bodies, and globular-shaped bodies.

The purpose of the SEM analyses was to identify changes in the morphology of the examined cells. The SEM images revealed a complex cell surface topography in all five cell lines (Figure 9 and Figure 10). Generally, the control cells were adherent and displayed a flattened-polygonal shape and typical morphological features, which included microvilli on their surface and other protrusions such as lamellipodia and filopodia. The adjacent cells were very often interconnected via processes in various arrangements. The formation of the microvilli not only depended on the cell line but also varied within particular samples. Many T986, MOGGCCM, and SW1783 cells had characteristic dense microvilli that were evenly distributed on the whole surface (Figure 9a,c and Figure 10a). The cells of the other lines were found to possess scarce microvilli (LN229) and almost no microvilli (LN-18) (Figure 9b and Figure 10b). These cells had a much smoother appearance in contrast to the T986, MOGGCCM, and SW1783 cells. Another morphological trait was the presence of lamellipodia and filopodia, especially at the lateral edges. The untreated control T98G cells showed the presence of parallel thin processes (filopodia) connecting the adjacent cells, whereas numerous lamellipodia were found in the MOGGCCM cells (Figure 9a and Figure 10a). The SW1783 cells exhibited single shorter and longer filopodia, and bundles of thin and long projections were detectable between some adjacent cells (Figure 10c). In contrast to the previously described cells, the LN cells occasionally had long filopodia without contact with adjacent cells. These cells were interconnected, although rarely, via lamellipodia (Figure 9b and Figure 10b).

When the T98G and MOGGCCM cells were treated with lensoside Aβ, many microvesicles were found at their edges and on the surface, and the arrangement of the microvilli changed to irregular clusters. A decrease in the number of interconnections between adjacent ins was observable as well. The cells (MOGGCCM) still had processes. In both LN229 and LN-18 cells, lensoside induced formation of not only microvesicles (at their edges and on the surface) but also ruffles. The SW1783 cells were weakly sensitive to lensoside Aβ. In these cells, microvilli were present, and the cells were connected via TNT-like processes (tunneling nanotubes) with vesicles. No long projections between adjacent cells were noted (Figure 9d–f and Figure 10c,d).

The sorafenib-treated T986, MOOGGCCM, and SW1783 cells exhibited loss of microvilli and long interconnections between adjacent cells. They had a smoother phenotype. The MOOGGCCM cells revealed the presence of rufflings and blebs under the influence of sorafenib. The LN229 cells were not sensitive to the sorafenib treatment (almost no changes were observed); oppositely, the cells of the LN-18 line had a globular shape and thin processes between them (Figure 9g–i and Figure 10e,f).

Huge alterations in morphology were found in all cell lines subjected to the combined treatment (lensoside Aβ plus sorafenib). A characteristic feature was the formation of globular-shape cells or cells with deformed bodies in all lines. The T98G cells exhibited loss of microvilli and formation of blebs on their surface. Almost no parallel cellular processes connecting neighboring cells were observed. The LN229 and LN-18 cells had ruffles and microvesicles on their surfaces. There were also cells with a multi-angular shape with lamellipodia and short filopodia. The combined treatment of the SW1783 cells caused formation of microvesicles at their edges. In the case of MOOGGCCM, there were also cells with long and branched filopodia, lamellipodia, and TNT-like linkages (Figure 9j–l and Figure 10g,h).

### 2.5. FTIR Spectroscopy of T98G, MOGGCCM, LN-18, LN229, and SW1783 Cells

**Figure 11:**
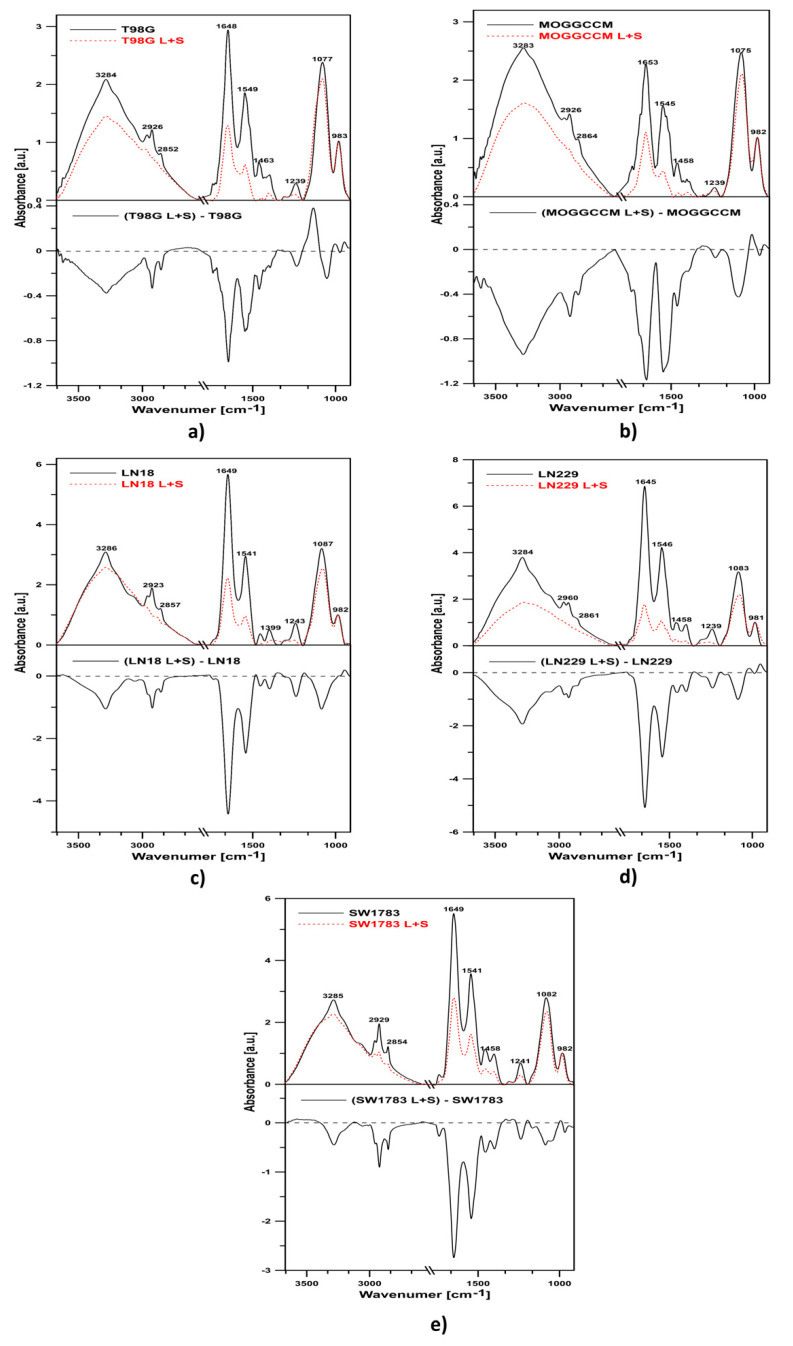
ATR-FTIR spectra of the MOGGCCM (**a**), T98G (**b**), LN229 (**c**), LN-18 (**d**), and SW1783 cells (**e**). The top panels of the graphs show spectra of the control cells (black solid line) and cells treated with a combination of lensoside Aβ and sorafenib (red dashed line) for 24 h. The lower panels present differential spectra of the cells.

The effect of the combined action of lensoside Aβ and sorafenib on the T98G, MOGGCCM, LN-18, LN229, and SW1783 cells was investigated with the use of Fourier-transform infrared absorption spectroscopy (FTIR). Figure 11 shows representative IR spectra of the cells in the region between 4000 and 970 cm^−1^ after 24-h treatment with the tested compounds.

The major absorbance peaks observed in the region between 4000 and 970 cm^−1^ are associated with the absorption modes of lipids, proteins, and nucleic acids. The band between 2800 and 3000 cm^−1^, with the maxima of the peaks at 2851 cm^−1^ and 2926 cm^−1^ as well as 2873 cm^−1^ and 2954 cm^−1^, represents symmetric and asymmetric C–H stretching vibrations of the CH_2_ and CH_3_ groups, respectively, mainly contained in cell lipids. The signal at ~1741 cm^−1^ corresponds to the C=O stretching vibrations of the ester carbonyl groups of phospholipids. The regions between 1700–1600 cm^−1^ and 1580–1510 cm^−1^ are characteristic for the amide I and amide II of protein, respectively. Absorptions between 1300 and 900 cm^−1^ are mainly attributed to phosphate associated with nucleic acids, DNA, RNA, and phospholipids. The signal at ~1460 cm^−1^ corresponds to the CH_2_ group deformation vibration (referred to as scissoring vibration). The bands at 1238 cm^−1^ and 1080 cm^−1^ represent antisymmetric and symmetric stretching of the PO_2_^−^ group in nucleic acid and phospholipids. The band at 970 cm^−1^ corresponds to antisymmetric N-(CH_3_)_3_ stretching vibrations.

**Figure 12:**
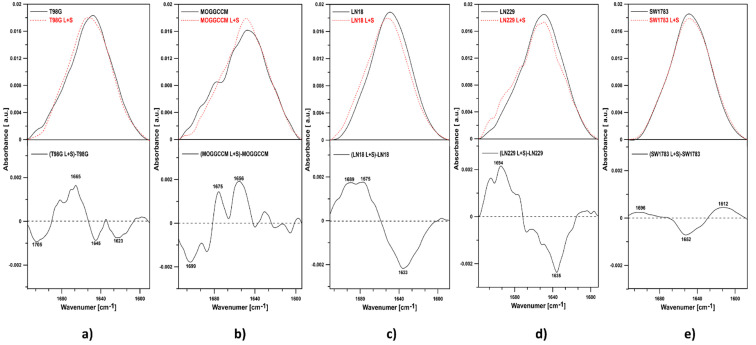
ATR-FTIR spectra of the amide I region (1700–1600 cm^−1^) of the control cells (upper panel, solid black line), of the cells incubated with lensoside Aβ and sorafenib for 24 h (upper panel, red, dashed line), and the differential spectra (lower panel) from (**a**) T98G, (**b**) MOGGCCM, (**c**) LN-18, (**d**) LN229, and (**e**) SW1783.

In the current study, notable spectral differences were found after the LAβ and sorafenib exposure, particularly in the 3300–3100 cm^−1^ (amide A), 3000–2800 cm^−1^ (lipids), 1700–1600 cm^−1^ (amide I), 1580–1510 cm^−1^ (amide II), and 1200–900 cm^−1^ regions in all the cell lines. The overall intensity of all the peaks showed significant changes relative to the control cells (Figure 11 and Figure 12).

In the differential spectra of all the tested cells, the presence of a negative band in the region of 3300–3100 cm^−1^, representing amide A, was observed. Cells treated with the combination of drugs showed a pronounced overall absorption decrease in the region between 2800 and 3000 cm^−1^, compared with the control cells (Figure 11). Moreover, all the cell lines revealed reduced intensity of the bands in the region between 900 and 1300 cm^−1^, compared with the control cells. Simultaneously, in the T98G line, the band corresponding to symmetric PO_2_^−^ stretching shifted towards higher frequencies (1077 cm^−1^). Such an effect is most probably related to breakage of hydrogen bonds.

As shown in Figure 11, negative bands in the amide I and II spectral region were noticed in all the cell lines after the combined treatment. This indicates a decreased relative protein concentration in the cells.

To determine the overall protein secondary structure in the cells, we analyzed the spectral bands in the amide I region (Figure 12). The bands in the amide I region represent different secondary structures of proteins, including antiparallel β-sheet (1675–1695 cm^−1^), turns (1660–1686 cm^−1^), unordered structures (1640–1648 cm^−1^), α-helix (1648–1660 cm^−1^), β-sheet (1625–1640 cm^−1^), and aggregated strands (1610–1628 cm^−1^) [28]. In the LN-18 and LN229 cells, there was an increase in antiparallel β-sheets and turns with a simultaneous decrease in β-sheets. In the SW1783 cells, a decrease in α-helices were observed. In turn, in the T98G line, there was an increase in the antiparallel β-sheets and turns and a decrease in the α-helices and β-sheets. Different results were obtained in the MOGGCCM cells. In these cells, an increase in turns, α-helices, and β-sheets and simultaneous reduction of aggregated stands were noted. Such results suggest biochemical changes in the cellular protein secondary structure after the treatment with both drugs simultaneously.

In the case of separate lensoside Aβ treatment (Figures S7 and S8), a clear decrease in protein concentration in regard to lipids was noticed in the MOGGCCM, LN229, and SW1783 cell lines. Tiny changes (almost no) were found in T98G and LN-18 cells. Analysis of the amide I region revealed no changes in the spectral profile of LN-18 and LN229 under the influence of LAβ. The biggest changes were noted for MOGGCCM and SW1783 cells. In MOGGCCM cells, unordered structures were created to the expense of parallel and antiparallel β-sheets. In SW1783 cells, there was an increase in antiparallel β-sheets, (probably pseudo β-sheets originating from aggregated strands). The T98G line showed a small increase in turns.

As for a single sorafenib application (Figures S9 and S10), the presence of negative bands in the region of 3300–3100 cm^−1^ (representing amide A) and in the region of 1700–1600 cm^−1^ and 1580–1510 cm^−1^, corresponding to the amide I and II spectral region, were observed. Such results indicate a decrease in the relative protein concentration in the examined cells. Analysis of the amide I region of all cell lines treated with sorafenib showed that in all cells a reduction in the β-sheet structures was noticed. At the same time, an increase in turns and loops was observed.

### 2.6. FLIM Microscopy of T98G Cells

**Figure 13:**
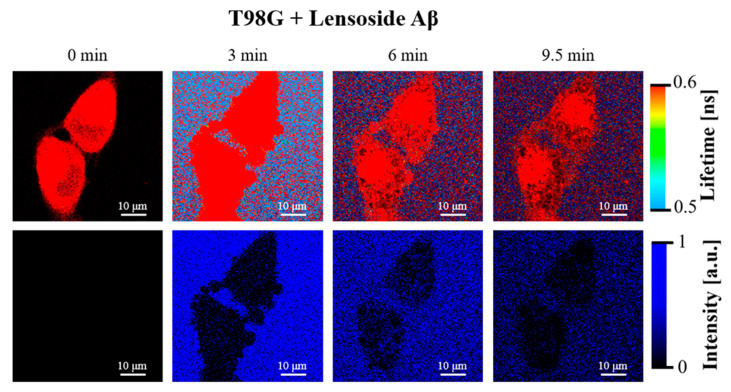
FLIM images of the T98G cells incubated with lensoside Aβ for the indicated different periods. The lower panel presents the same images created solely on the fluorescence lifetime component of 0.01 ns, representing selectively lensoside Aβ and showing its distribution in the examined system. See also Figure S3 for images of the other cells. Note a pronounced cell membrane deformation in the first 3 min of incubation.

**Figure 14:**
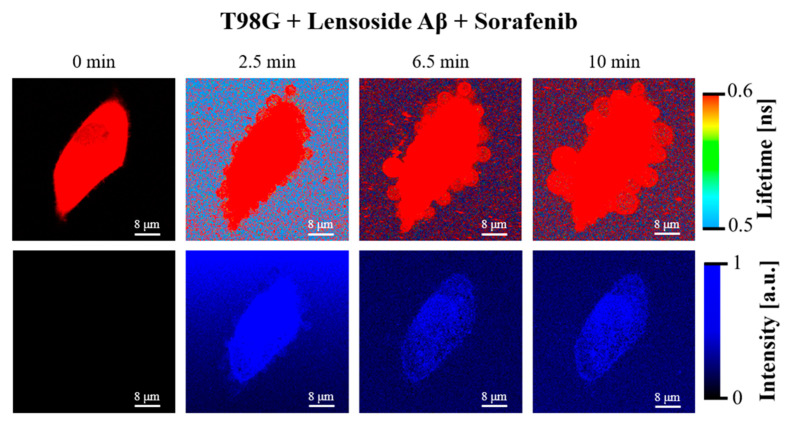
FLIM images of T98G cells incubated with lensoside Aβ and sorafenib for the indicated different periods. The lower panel presents the same images created solely on the fluorescence lifetime component of 0.01 ns, representing selectively lensoside Aβ and showing its distribution in the examined system. See also Figure S4 for images of the other cells. Note a pronounced cell membrane deformation, formation of extracellular vesicles, and diffusion of lensoside Aβ into the cell.

The T98G cells were imaged using the fluorescence lifetime imaging microscopy (FLIM) technique with the application of the 375 nm laser, suitable to excite lensoside Aβ. Fluorescence emission of lensoside Aβ is characterized by a virtually single fluorescence lifetime of 0.01 ns, and it was applied to monitor the interaction of lensoside Aβ with the T98G cells (Figure 13 and Figure 14).

As can be seen in Figure 13, the exposure of the cells to lensoside Aβ resulted in relatively fast deformation of the cell membranes and gradual penetration of the flavonoid into the cell (see also Figure S3). The first effect was significantly enhanced upon the presence of sorafenib (Figure 14 and Figure S4). In such a case, the cell membrane reacted to the presence of the drugs by formation of extra-membranous (extracellular) structures that were gradually detached from the cells. Such an effect was observed upon the exposure of the T98G cells to sorafenib alone (see Figure S5). On the other hand, the morphological analysis showed that the “protective” mechanism of the T98G cells, consisting of the elimination of sorafenib molecules via formation of extracellular vesicles (Figure S5), was apparently not as effective under the additional presence of lensoside Aβ (Figure 14). In our opinion, such a mechanism can be responsible for the synergistic effect of lensoside Aβ and sorafenib against T98G cells. For some cells, diffusion of lensoside Aβ into the cell was observed.

## 3. Discussion

It is well known that natural bioactive compounds may act in synergy with drugs used in anticancer therapy [27]. Our present pioneer study has revealed that sorafenib with lensoside Aβ is a very promising combination, effectively eliminating anaplastic astrocytoma and glioblastoma multiforme cells via apoptosis. This is a valuable observation, given the extremely high resistance of gliomas to treatment. The median survival in patients with anaplastic astrocytoma is about 30 months, while it is less than 15 months in the case of glioblastoma multiforme under standard care treatment [21,22,23]. The background of such a phenomenon should be sought at the molecular level, as malignant gliomas develop several mechanisms responsible for blocking programmed cell death induction while molecular survival pathways are overstimulated [24,29]. Recent publications indicate that most gliomas display upregulated Raf, which is essential for activation of the pro-survival mitogen-activated Ras–Raf–MEK–ERK protein pathway [30,31,32]. One of the inhibitors of this signal transmission is sorafenib, which is indicated as a standard treatment for advanced renal cell carcinoma (RCC), unresectable hepatocellular carcinomas (HCC), and thyroid cancer. Attempts to use this compound in the treatment of gliomas have also been made. Intracranial application of sorafenib caused inhibition of cell proliferation, reduction of angiogenesis, and induction of autophagy and apoptosis. Systemic administration of sorafenib was well tolerated and the drug crossed the blood–brain barrier effectively [26,33]. On the other hand, disappointing effects were achieved in some cases [25,26]. Generally, it has been demonstrated in various tumor cell lines that sorafenib induces apoptosis [34] but may also initiate autophagy [26]. Our experiments have confirmed these observations, revealing that sorafenib itself may induce both types of death and the process is cell-line specific. In three anaplastic astrocytoma (MOGGCCM, LN229, and SW1783) cell lines and glioblastoma multiforme LN-18 cells, apoptosis was dominant, but the effect was generally not satisfactory. In the T98G glioblastoma cells, autophagy was observed most frequently.

Our previous experiments showed that the antiglioma potential of sorafenib could be increased by the flavonoid quercetin [27]. Lensoside Aβ (LAβ) is a quercetin glycoside [16]. According to some reports suggesting that glycosylation may be an effective approach for enhancing the pharmaceutical properties of different natural products [35], we decided to combine lensoside Aβ with sorafenib for the first time and to estimate their antiglioma potential. Our results indicate that lensoside Aβ alone initiates mainly apoptosis in most of cancer cell lines studied, but the percent of dead cells was cancer type and grade specific. Interestingly, the flavonoid caused significant changes in the percent of apoptotic cells only in the anaplastic astrocytoma lines, while the level of apoptosis in the glioblastoma multiforme cells was only slightly higher than that observed in the non-treated control cells. No autophagy or necrosis was noticed. After the treatment with both drugs, the levels of apoptosis were higher than that observed after the single sorafenib or lensoside Aβ applications, and the effect was synergistic. At the molecular level, this was confirmed by the reduced mitochondrial membrane potential and the elevated level of active caspase 3. No autophagy was observed, not even in the T98G cells, where sorafenib alone initiated this type of death most frequently. This observation is very interesting in the light of studies demonstrating that autophagy may represent a protective mechanism, helping cancer cells to eliminate toxic particles and, as a consequence, increase their survival. Therefore, redirecting cancer cells to enter the apoptotic pathway would be beneficial [36,37]. It seems that lensoside Aβ may play a key role in elimination of glioma cells via apoptosis rather than autophagy. At the molecular level, the balance between caspase 3 and beclin 1 may have a critical role in such diversity. It has also been shown that activation of caspase 3 inhibits autophagosome formation and may block beclin 1 activity or even cleave the protein [38,39]. In the T98G cells, the level of caspase 3 after the lensoside Aβ treatment was diminished, in contrast to the simultaneous drug treatment, where a very significant increase in the level of protein was noticed. In our study, sorafenib and lensoside Aβ applied alone or in combination decreased the level of Raf kinase expression, thereby upregulating the Ras–Raf–MEK–ERK pathway. This indicates that decreased signal transmission through the Ras–Raf–MEK– ERK cascade increases the sensitivity of glioma cells to death upon sorafenib and lensoside Aβ treatment and stimulates mainly apoptosis.

This is also in agreement with data obtained with the FTIR technique, which facilitates monitoring global changes in the protein secondary structure and apoptosis identification. In the current investigations, pronounced effects were found in the regions representing secondary structures, but the changes in the spectral profiles of proteins under the influence of lensoside Aβ with sorafenib were cell-line specific. The MOGGCCM cells exhibited an increase in turns and β-sheets, which is associated with apoptosis [40]. In turn, an increase in antiparallel β-sheets was observed in LN-18 and LN229, while a decrease in α-helices was noted in the T98G and SW1783 lines. Generally, the relative protein concentration in all the examined cells was reduced, as a decrease in the oscillator strengths of amide I and II was visible in the ATR-FTIR spectra. Conformational changes in protein structures might be coupled with proteolysis exerted by apoptotic caspases. Alterations in the quality and quantity of proteins might also result from modulation of the chaperone activity or proteasome function. Consequently, the overall folding can be affected, which was visible in the vibrational frequencies specific for peptide bonds. One cannot exclude that the biochemical changes in the cellular protein secondary structure after the treatment with lensoside Aβ with sorafenib might result from interactions of the examined drugs with proteins and the destabilization thereof by breakage of the hydrogen bonds [41]. The current investigation also revealed important changes in the region 1300–900 cm^−1^, referred to the phosphates associated with DNA and phospholipids [42]. The decrease in DNA absorbance, considered as an apoptosis biomarker, can be explained as a result of the conformational changes in DNA under the influence of both drugs [41,43].

An additional crucial aspect of apoptosis is the loss of asymmetric distribution of plasma membranes. In our study, a pronounced decrease in overall absorption in the region 2800–3000 cm^−1^ (characteristic for fatty acyl chains of all cellular lipids) was observed. In this region, the maxima of the peaks, at 2851 cm^−1^ and 2926 cm^−1^ as well as 2873 cm^−1^ and 2954 cm^−1^, represent symmetric and asymmetric C–H stretching vibrations of the CH_2_ and CH_3_ groups, respectively. The negative band intensities in the difference spectra observed in the present study may reflect structural changes in phospholipids [42].

The morphological observations of glioma cells using the SEM technique provided further evidence for the beneficial effect of lensoside Aβ in increasing the anti-glioma potential of sorafenib. Glioma cells are characterized by a complex surface topography with microvilli (very dense in some studied lines), filopodia, and lamellipodia, as well as long interconnections between adjacent cells, sometimes arranged in parallel processes or in bundles. These membrane protrusions allow these cells to infiltrate surrounding tissue. Microvilli and filopodia are involved in migration and determine the invasion abilities of the cells. This phenomenon may be correlated with many cytokine receptors located at these structures, e.g., HER2/neu (receptor tyrosine-protein kinase erbB-2), EphA2 (ephrin type-A receptor 2), IL-13 receptor α2 (interleukin-13 receptor alpha 2), EGFR (epidermal growth factor receptor), and PDGFR (platelet-derived growth factor receptor) [44,45]. In our experiment, all the control cells were adherent to the surface but, after the treatment with lensoside Aβ and sorafenib, they became globular with characteristic blebs. Additionally, the decrease in the interconnections, loss/decrease in the number microvilli and filipodia, and the changes in the topography may indicate a less invasive phenotype of the glioma cells after the treatment with both drugs. Taking into consideration the exceptional infiltrating and migratory nature of gliomas [21], such results might be very beneficial in terms of anticancer treatment. Moreover, rufflings and microvesicles on the edges and surface of the cells were noticed. These observations are in a good correlation with our FLIM data. For this experiment, the representative T98G cell line was chosen, since autophagy was observed upon the separate sorafenib incubation only in this line, besides the strong apoptosis after lensoside Aβ and sorafenib action. The FLIM technique revealed the presence of extramembranous vesicles attached to the cells upon the separate lensoside Aβ treatment and in combination with sorafenib. Such morphological changes may be a response to the flavonoid gradually penetrating into the cells, as LAβ is known for its intercalation and interaction with membranes, changing their structure and properties as a consequence [18]. Interestingly, this effect was accelerated by the additional sorafenib incubation. The separate treatment of glioma cells with sorafenib resulted in formation of vesicles, which detached from the cells with time. This is in agreement with our former observations of T98G cells incubated with sorafenib, where vesicles were concentrated at the edges of cells and were not dispersed throughout the cytoplasm. The authenticity of the peripheral vacuoles as autophagic ones was confirmed by the localization of beclin 1 therein [27]. The cause of such an unusual distribution is unknown, but it may be a part of the protective mechanism of elimination of potentially fatal particles, in this case sorafenib, from cells. The FLIM analysis showed that the additional lensoside Aβ treatment blocked the detachment of vesicles, which remained connected to the cell. This is another confirmation that lensoside Aβ strengthens the action of sorafenib and increases the efficiency of both drugs against glioma cells.

## 4. Materials and Methods

### 4.1. Cells and Culture Conditions

Human glioblastoma multiforme cells (T98G, ECACC 92090213) and human anaplastic astrocytoma cells (MOGGCCM, ECACC 86022702) were grown in a 3:1 mixture of Dulbecco’s Modified Eagle Medium (DMEM) and Ham’s nutrient mixture F-12 (Corning, Tewksbury, MA USA) supplemented with 10% fetal bovine serum (FBS, Corning). LN-18 and LN229 (human glioblastoma, ATCC, CRL-2610, and CRL-2611) were cultured in DMEM with 5% FBS, and SW1783 (human anaplastic astrocytoma, ATCC, HTB-13) cells were cultured in DMEM with 10% FBS. All cell lines were supplemented with 100 μg/mL of penicillin and streptomycin. The cultures were kept at 37 °C in a humidified atmosphere of 95% air and 5% CO_2_.

To confirm the effect of lensoside Aβ on normal cells, the OLN-93 oligodendrocyte cell line (obtained from Department of Neonatology, Charite, Campus Virchow Klinikum, Humboldt University Berlin) was used (Supplementary Data Figure S2). OLN-93 retains the morphological features of O-2A progenitors or immature oligodendrocytes and its antigenic properties resemble primary oligodendrocytes in culture. The culture medium consisted of a 1:1 mixture of DMEM and the nutrient mixture Ham F-12 supplemented with 10% fetal bovine serum (FBS), 100 IU/mL penicillin, and 100 μg/mL streptomycin. Cells were grown at 37 °C in a humidified atmosphere of 95% air and 5% CO_2_.

### 4.2. Lensoside Aβ (LAβ) Isolation

Lensoside Aβ (see Figure S6 for chemical structure) was isolated from aerial parts of lentil according to a procedure described in detail previously [16]. It was dissolved in ethanol (Merck, Darmstadt, Germany) and used in the studies. The solution was kept in the dark.

### 4.3. Drug Treatment

Sorafenib (Nexavar, BAY 43-9006, Bayer HealthCare AG, Leverkusen, Germany see Figure S6 for chemical structure) was dissolved in DMSO to the final concentration of 1 µM in the case of the MOGGCCM and T98G cells, which was chosen based on our previous experiments [27]. The LN229, LN-18, and SW1783 lines were incubated with sorafenib at the concentration range of 0–5 µM, and incubation with 1 µM of the drug was chosen for further experiments. The concentration of lensoside Aβ, i.e., 50 µM in the MOGGCCM, T98G, LN229, and SW1783 cells or 25 µM in the LN-18 cells, was established experimentally. The concentrations of both drugs used in combination were established experimentally and confirmed with the Chou–Talalay method. The cancer cells were treated with the flavonoid or with sorafenib separately or in combination for 24 h (incubation time estimated experimentally). As controls, the cell lines were incubated only with 0.01% of DMSO (without sorafenib and lensoside Aβ), which had no effect on cell viability, as shown in previous experiments.

### 4.4. Microscopic Detection of Apoptosis, Autophagy, and Necrosis with Fluorochromes

For identification of apoptosis and necrosis in the MOGGCCM, T98G, LN229, LN-18, SW1783 and normal OLN-93 cells, a staining method with Hoechst 33342 (Sigma, St. Louis, MO, USA) and propidium iodide (Sigma, St. Louis, MO, USA) was chosen, as described previously [19,20,27]. In the case of autophagy, staining with acridine orange (AO) to detect typical acidic vesicular organelles (AVOs) was performed. A fluorescence microscope (Nikon E–800, objective 40×/0.75, camera Nikon D-200) was used for morphological analysis of dead cells. Cells exhibiting blue fluorescent nuclei (fragmented or/and with condensed chromatin) after Hoechst 33342 staining were interpreted as apoptotic. Cells exhibiting pink fluorescent nuclei upon propidium iodide were interpreted as necrotic. Typical AO-positive cells exhibiting granular discretion of AVOs in the cytoplasm were interpreted as autophagic. At least 1000 cells in randomly selected microscopic fields were counted under the microscope. Each experiment was conducted in triplicate.

### 4.5. Cell Death Detection by Flow Cytometry

The Annexin V-FITC apoptosis detection kit (Sigma, St. Louis, MO, USA) was used for identification of apoptosis and necrosis by flow cytometry. The samples were prepared according to the manufacturer’s protocol. Briefly, the control and drug-treated cells were incubated with 5 μL of Annexin V-FITC and 10 μL of propidium iodide for 10 min. Immediately after the staining, the cells were analyzed with the FacsCanto instrument (Becton–Dickinson, San Jose, CA, USA). In total, 30,000 events were acquired and analyzed using FacsDiva software. Cells that were in the early apoptotic process were stained only by Annexin V-FITC; late apoptotic cells were stained by both fluorochromes. Cells stained only by propidium iodide were interpreted as necrotic. Live cells showed no staining by either propidium iodide or Annexin V-FITC. Each experiment was performed in triplicate.

### 4.6. Detection of Mitochondrial Membrane Potential by Flow Cytometry

For the mitochondrial membrane potential (∆ψ_m_) (MMP) analysis, staining with fluorochrome 3,3′-dihexyloxacarbocyanine iodide (DiOC_6_(3)) was chosen according to the method described previously [27]. The control and drug-treated cells were incubated with fluorochrome at the final concentration of 50 nM for 20 min at 37 °C in the dark, washed three times with PBS, and analyzed with the FacsCanto instrument (Becton Dickinson, San Jose, CA, USA). Each experiment was performed in triplicate.

### 4.7. Immunoblotting

Whole cell extracts were prepared by lysing cells in hot buffer containing 125 mM Tris-HCl pH 6.8, 4% SDS, 10% glycerol, and 100 mM dithiothreitol (DTT). The protein concentration was measured with the Bradford method [46]. In total, 80 µg of proteins were separated by 10% SDS-PAGE [47] and transferred onto Immmobilon P membranes (Sigma). After blocking with 3% low fat milk for 1 h, the membranes were incubated overnight with primary antibodies: mouse anti-Beclin 1 (BECN1 (E-8): sc-48341, Santa Cruz Biotechnology, 1:500), rabbit anti-caspase 3 (caspase-3 p17 (H-60): sc-98785 (SantaCruz Biotechnology dilution 1:1000), and anti-Raf (A-Raf (C-20): sc-408, Santa Cruz Biotechnology, 1:500). After three washes with PBS enriched with 0.05% Triton X-100 (Sigma), the membranes were incubated with secondary antibodies (mouse or rabbit, Sigma, dilution 1:30,000) conjugated with alkaline phosphatase (AP) for 2 h. Proteins were detected with AP substrates: 5-bromo-4-chloro-3-indolylphosphate (BCIP) and nitro-blue tetrazolium (NBT) (Sigma) in N,N-dimethylformamide (DMF, Sigma). The results obtained were analyzed qualitatively based on the band thickness, width, and color depth. The quantitative analysis of protein bands was performed using the Bio-Profil Bio-1D Windows Application V.99.03 program. The data were normalized relative to β-actin (Sigma, working dilution 1:2000). Three independent experiments were performed. The whole western blot figures can be found in Figure S1.

### 4.8. Fourier-Transform Infrared Absorption Spectroscopy (FTIR) of Cells

The cells were incubated for 24 h at 37 °C alone (control) and in the presence of LAβ at a concentration of 50 µM (25 µM for the LN-18 line) and sorafenib 1 µM, alone and in combination. Next, the medium was removed from the flasks, and monolayer cells were rinsed with PBS, gently scraped using cell scrapers, and then suspended in PBS buffer with 5% addition of D_2_O (100 mM, pH 7.4). Infrared absorption spectra of the cells (control and incubated with the examined drug or drug mixture) were recorded using a Fourier-transform infrared absorption spectrometer equipped with an attenuated total reflection setup (ATR-FTIR) [48]. The spectra were recorded with a Nicolet iS50R device from Thermo Scientific, USA. Continuous purging with argon was employed during the measurements. After gentle centrifugation, the samples were deposited on the ATR crystal element by evaporation from PBS buffer with addition of D_2_O (pH 7.4). An internal reflection element (diamond prism) was used as an attenuated total reflection element (ATR). Typically, 10 scans were collected, Fourier-transformed, and averaged for each measurement. Absorption spectra at a resolution of one data point every 2 cm^−1^ were obtained in the region between 4000 and 400 cm^−1^, using a clean crystal as the background. All experiments were done at 21 °C. Spectral analysis was performed with Grams AI software from ThermoGalactic (Waltham, MA USA).

### 4.9. Scanning Electron Microscopy (SEM) of Cells

Scanning electron microscopy (TESCAN vega 3 LMU, Brno, Czech Republic) analyses were performed to investigate the morphology of the cells. First, the cells were incubated at 37 °C for 24 h without (control) or with the drugs alone or in combination (LAβ plus sorafenib) at a concentration of 50 µM (or 25 µM for the LN18 line of LAβ) and 1 µM of sorafenib. Next, the cells were fixed with 4% glutaraldehyde in 0.1 M cacodylate buffer (pH 7.2) for 2 h at 4 °C and the samples were rinsed with 0.1 M cacodylate buffer (pH 7.2). After washing, post-fixation was performed with freshly prepared 1% osmium tetroxide at 4 °C for 1 h. The subsequent rinsing was carried out with the use of 0.1 M cacodylate buffer (pH 7.2). The following step was dehydration in a series of ethanol gradients: 30%, 50%, 70%, 90%, and 100% (each for 10 min). Eventually, the samples were mounted on stubs and coated with gold in an Emitech K550X Sputter Coater. The specimens were analyzed in a secondary electron mode [49].

### 4.10. Fluorescence Lifetime Imaging Microscopy (FLIM)

The fluorescence emission spectrum and fluorescence intensity decay of lensoside Aβ were carried out using a FluoTime 300 spectrometer (PicoQuant GmbH). All the emission data were obtained using a 375 nm laser operating at a 20 MHz repetition rate. The fluorescence intensity decay was recorded for the emission maximum at 465 nm with a 4 ps resolution time using a 405 nm-long wavelength-pass filter (Chroma-AHF Analysentechnik). The fluorescence emission decay was fitted with four exponential components according to the formula:

$$I\left(t\right)=\underset{i}{\sum }\alpha_{i}e^{\frac{-t}{\tau_{i}}}$$

were $\alpha_{i}$ is the relative amplitude of the component and $\tau_{i}$ is the decay time.

Microscopy data were recorded on a MicroTime 200 confocal system (from PicoQuant GmbH), combined with an Olympus IX71 inverted microscope. The cell samples were excited with a 375 nm pulsed laser with a 10 MHz repetition rate and a 16 ps resolution time. A silicon-immersed objective (Olympus NA 1.3, 60×) was used in the experiments. The observation was performed in the confocal mode using a 50 μm in diameter pinhole and with a 430 nm-long wavelength-pass filter (Chroma-AHF Analysentechnik). The detection system was based on Excelitas Single Photon Avalanche Diodes. Fluorescence lifetime data were analyzed using SymPhoTime 64 v. 2.6 software (PiqoQuant) [50].

### 4.11. Chou–Talalay Method

The combination index (CI) and the dose reduction index (DRI) were calculated with the method developed by Chou and Talalay with Compusyn software, using the original data of programmed cell death induction in the MOGGCCM and T98G cells upon sorafenib or lensoside Aβ treatment. CI < 1, CI = 1, and CI > 1 indicate a synergistic, additive, and antagonistic effect, respectively. The DRI represents the fold reduction of compounds as a result of the synergistic combination compared to the concentration of the drug alone required to reach the same effect.

### 4.12. Statistical Analysis

A one-way ANOVA test followed by Dunnett’s multiple comparison analysis was used for statistical evaluation. A *p* < 0.05, calculated from data presented as the mean ± standard deviation (SD), was taken as the criterion of significance.

## 5. Conclusions

In summary, we have demonstrated for the first time that lensoside Aβ intensifies the anticancer potential of sorafenib, synergistically enhancing the susceptibility of human glioblastoma multiforme and anaplastic astrocytoma cells to apoptosis initiation. This could be correlated with the previously confirmed ability of the flavonoid to interact with membrane structures [18], which, as a consequence, could lead to morphological changes typical for programmed death. It also seems that the Ras–Raf–MEK–ERK pathway may play a key role in this process. Our results have demonstrated that sorafenib administered with lensoside Aβ seems to be a potent and promising combination that might be useful in glioma therapy.

## Data Availability

The data presented in this study are available from the corresponding author.

## Supplementary Materials

The following are available online at https://www.mdpi.com/article/10.3390/cancers13112637/s1, Figure S1: The effect of lensoside Aβ and sorafenib on normal cells, Figure S2: FLIM images of T98G cells incubated with lensoside Aβ, Figure S3: FLIM images of T98G cells incubated with lensoside Aβ and sorafenib, Figure S4: FLIM images of T98G cells incubated with sorafenib. Figure S5: FLIM images of T98G cells incubated with sorafenib for the indicated different periods, Figure S6: Chemical structure of sorafenib and lensoside Aβ, Figure S7: Representative ATR-FTIR absorption spectra, Figure S8: ATR-FTIR spectra at the region 1700–1600 cm^−^^1^, Figure S9: Efect of sorafenib applied alone, Figure S10: The amide I region (1700–1600 cm^−1^)
