# Supplementary material for: Lensoside Aβ as an Adjuvant to the Anti-Glioma Potential of Sorafenib
Source: Cancers (Basel). 2021 May 27;13(11):2637. doi: 10.3390/cancers13112637 (PMC8198162; doi:10.3390/cancers13112637)
Supplement: Supplementary file 1 [file cancers-13-02637-s001.zip › cancers-1222271-SI.pdf]

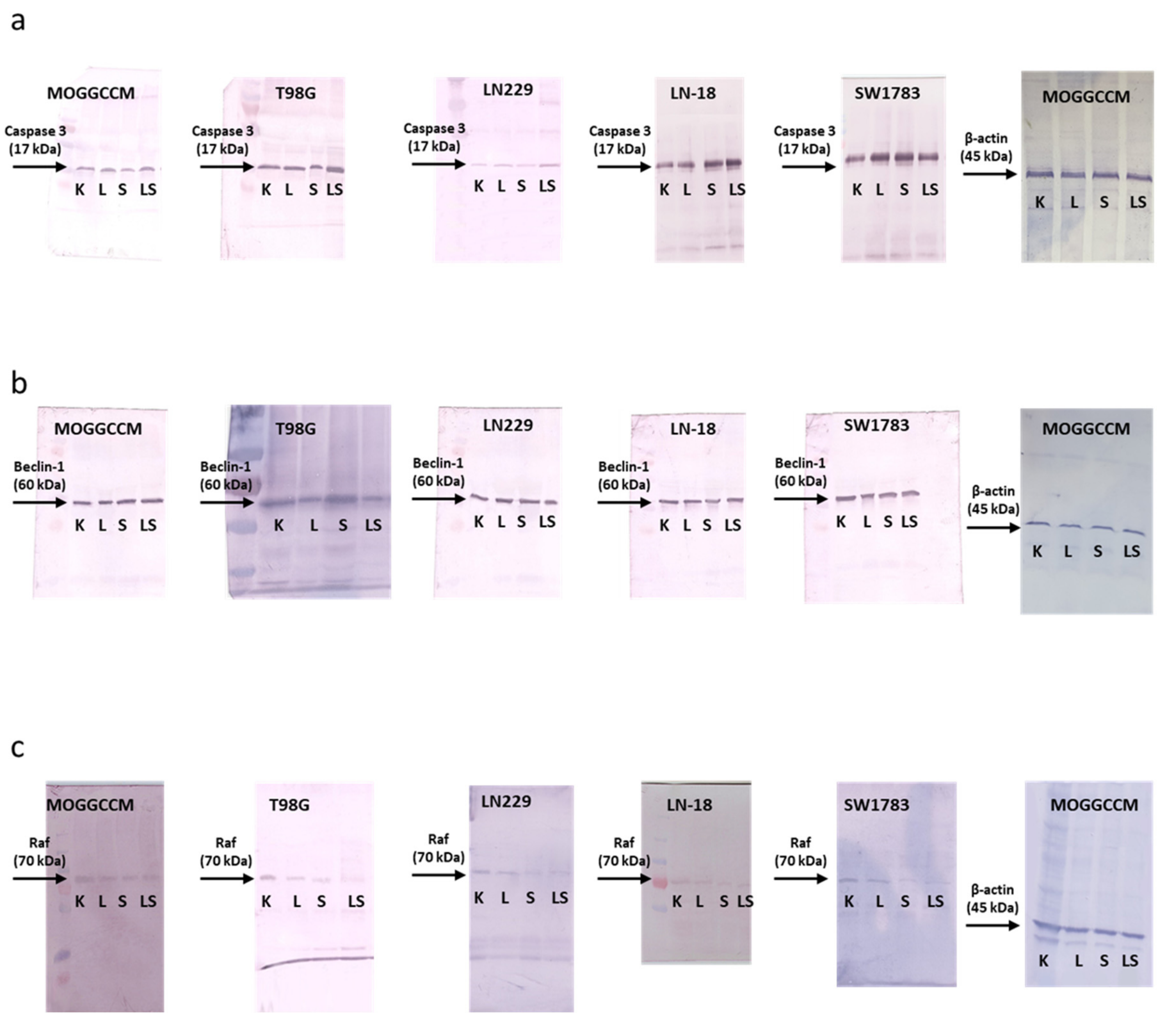

Figure S1. Representative, original Western blot membranes presenting the level of caspase 3 (a), beclin 1 (b) and Raf (c) in MOGGCCM, T98G, LN229, LN-18 and SW1783 cells as well as β-actin in MOGGCCM as representative loading control.

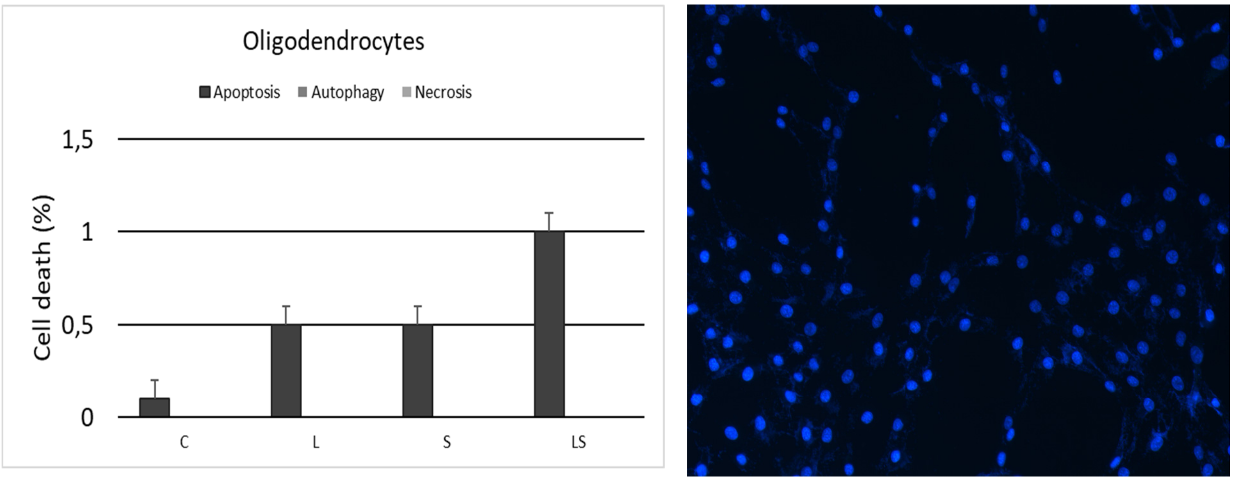

Figure S2. The effect of lensoside Aβ (L) and sorafenib (S) on the level of apoptosis, autophagy, necrosis identified microscopically after staining with Hoechst 33342, acridine orange and propidium iodide respectively in OLN-93 cells with the representative picture of cells after LS treatment (only living cells visible) . C-control

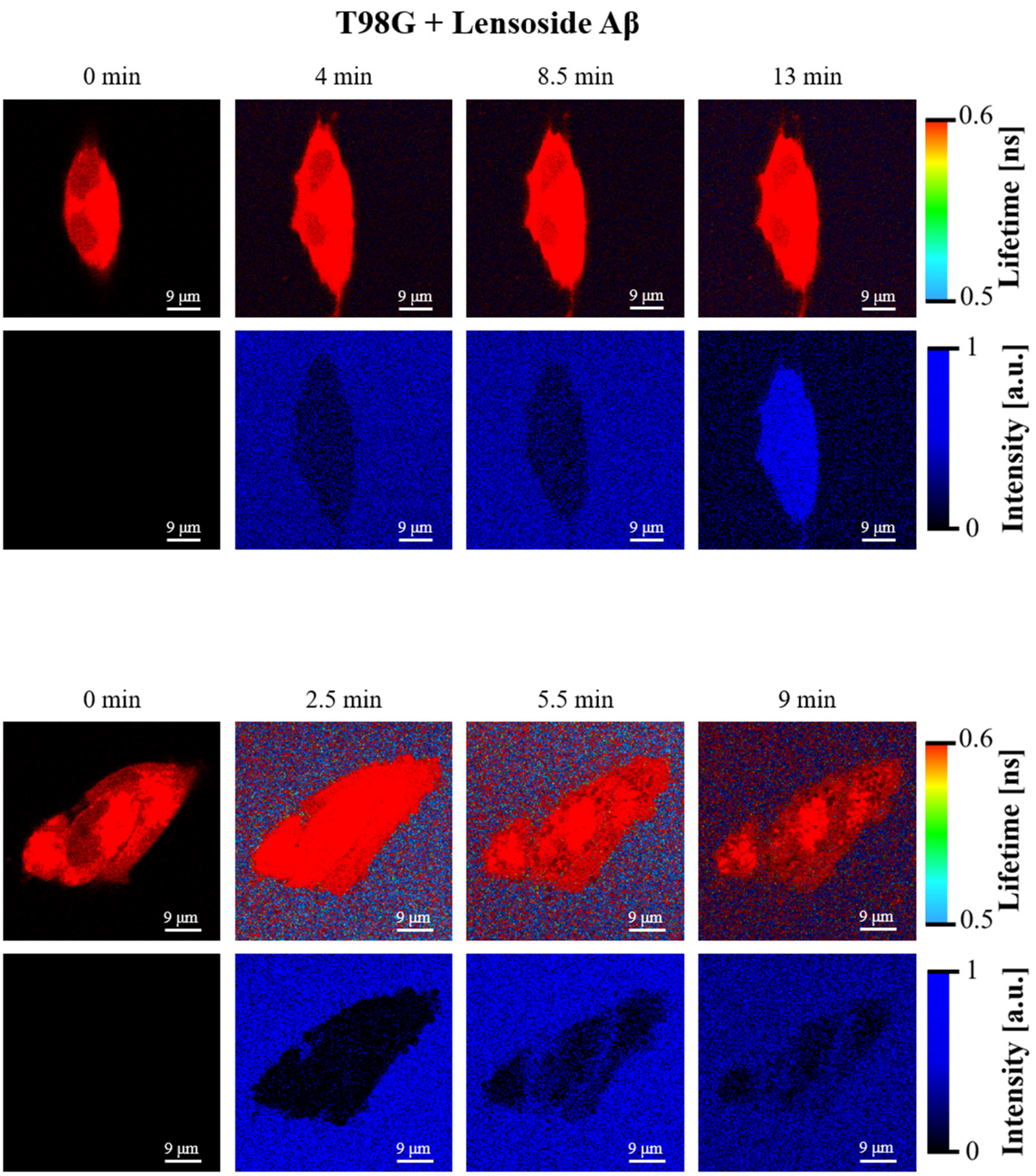

Figure S3. FLIM images of T98G cells incubated with lensoside Aβ for the indicated different periods. The lower panels of the two sets present the same images created solely on the fluorescence lifetime component of 0.01 ns representing selectively lensoside Aβ and showing its distribution in the examined system.

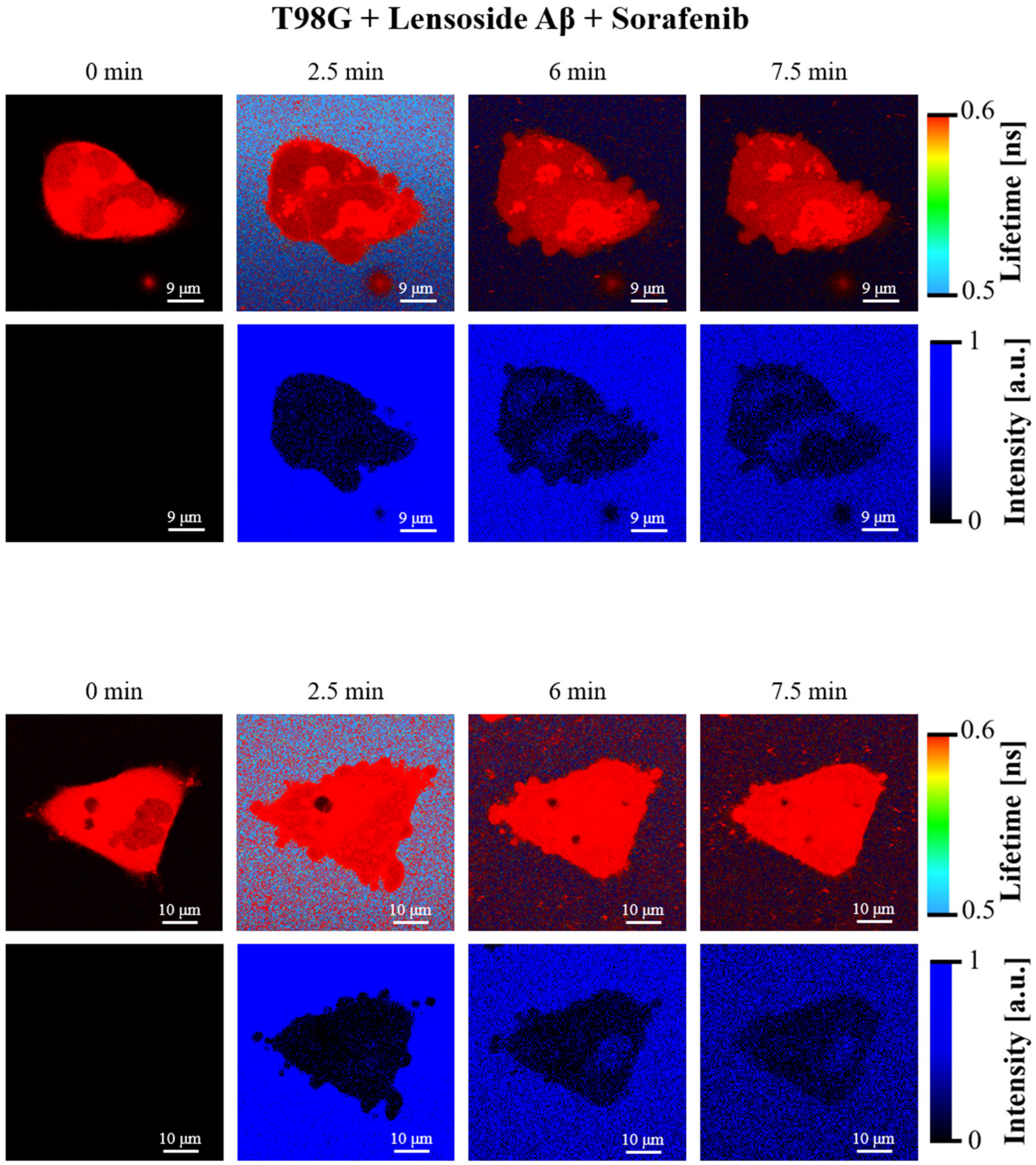

Figure S4. FLIM images of T98G cells incubated with lensoside Aβ and sorafenib for the indicated different periods. The lower panels of two sets present the same images created solely on the fluorescence lifetime component of 0.01 ns representing selectively lensoside Aβ and showing its distribution in the examined system.

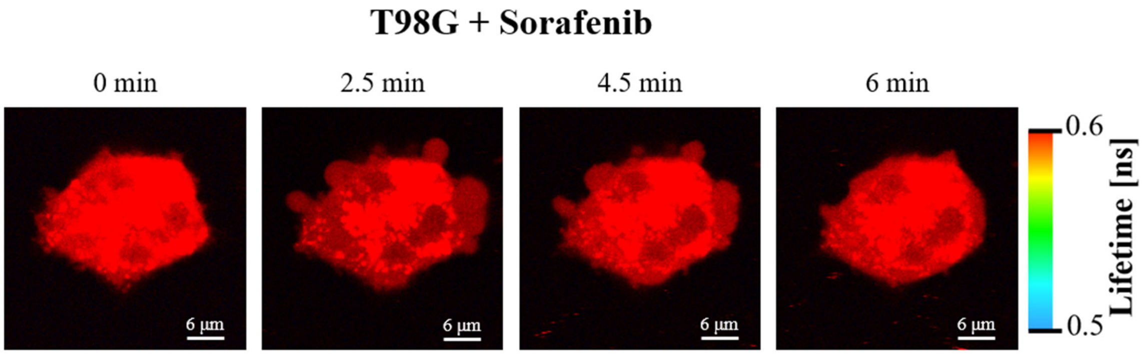

Figure S5. FLIM images of T98G cells incubated with sorafenib for the indicated different periods.

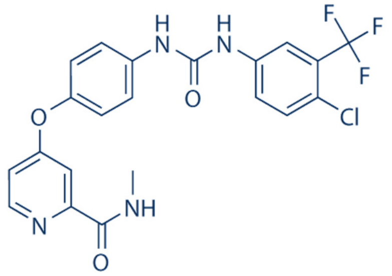

sorafenib

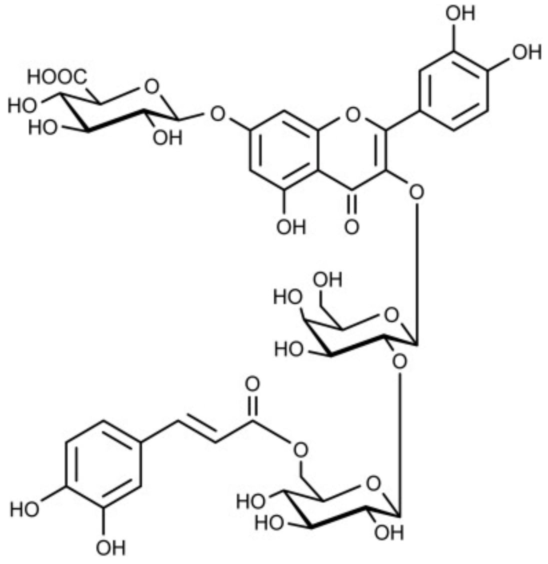

lensoside Aβ

Figure S6. Chemical structure of sorafenib and lensoside Aβ

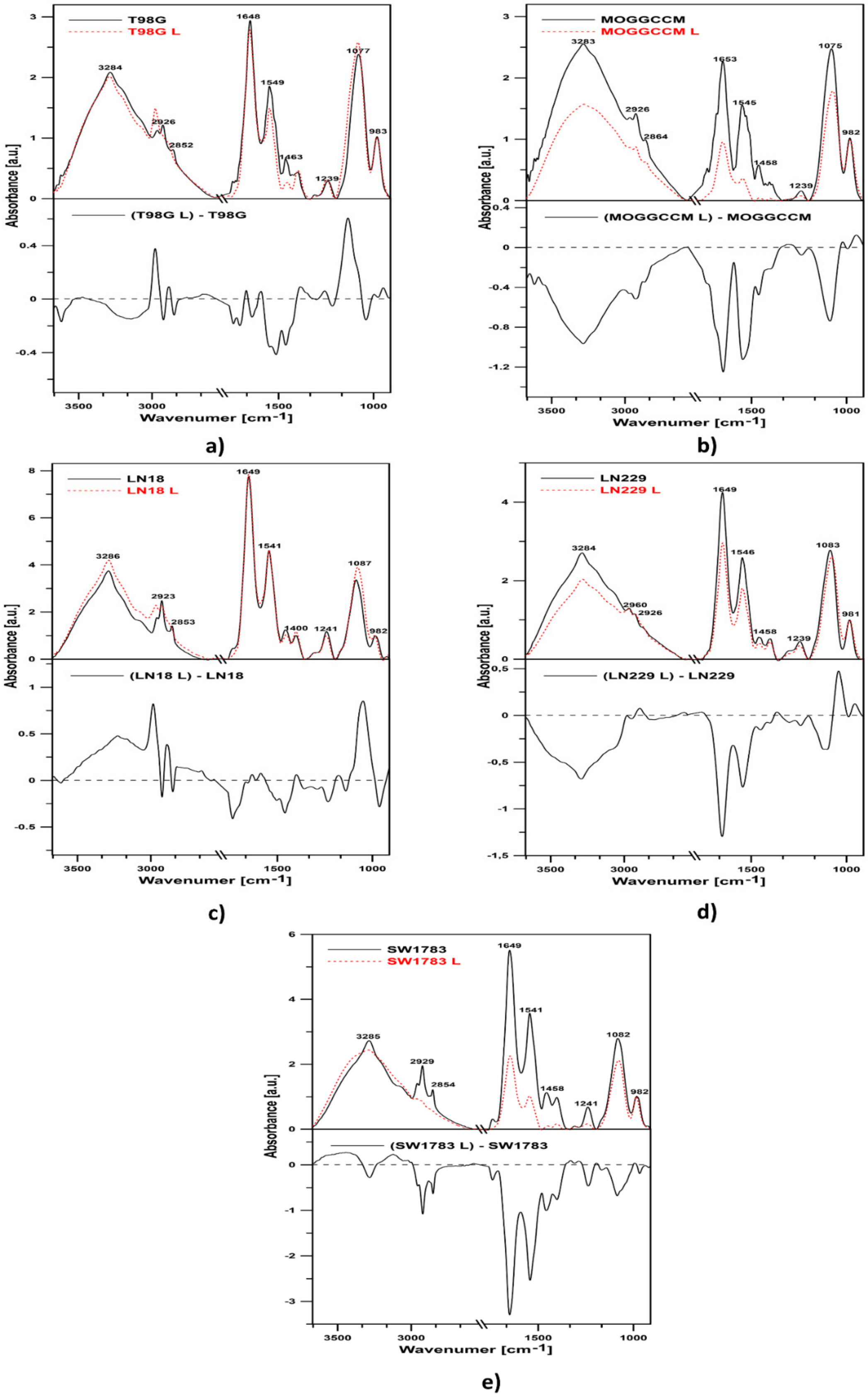

Figure S7. Representative ATR-FTIR absorption spectra of a) T98G, b) MOGGCCM , c) LN-18, d) LN229 and e) SW1783 cells in the spectra region 3600-900 cm^{-1}. The top panels spectra of control cells (black solid line) and

cells after 24 hour incubation with lensoside Aβ (red, dashed line) are shown. The lower panels present differential spectra of examined cells.

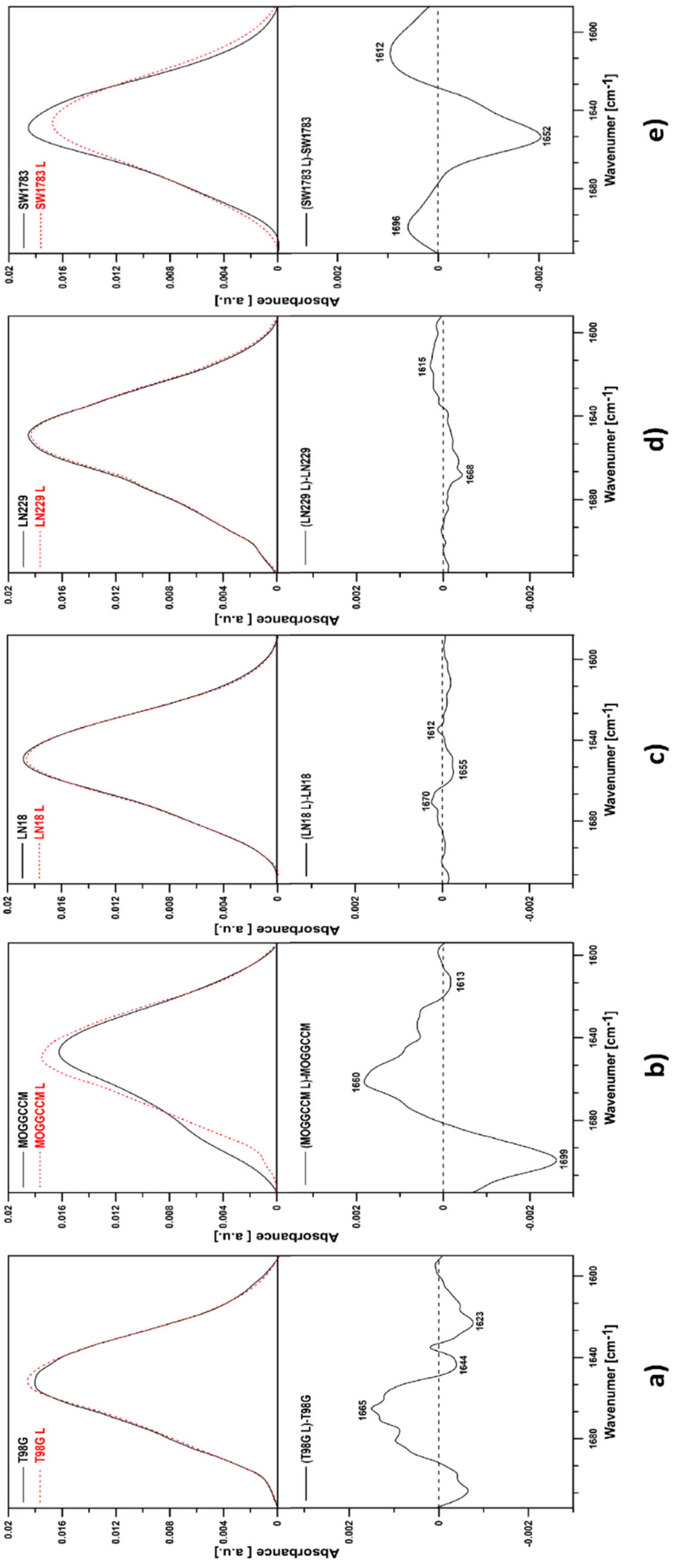

Figure S8. ATR-FTIR spectra at the region 1700-1600 cm^{-1} of control cells (upper panel, solid black line), cells treated with lensoside Aβ for 24 hours (upper panel, red, dashed line) and diffrential spectra (lower panel) from a) T98G, b) MOGGCCM, c) LN-18, d) LN229 and e) SW1783 line.

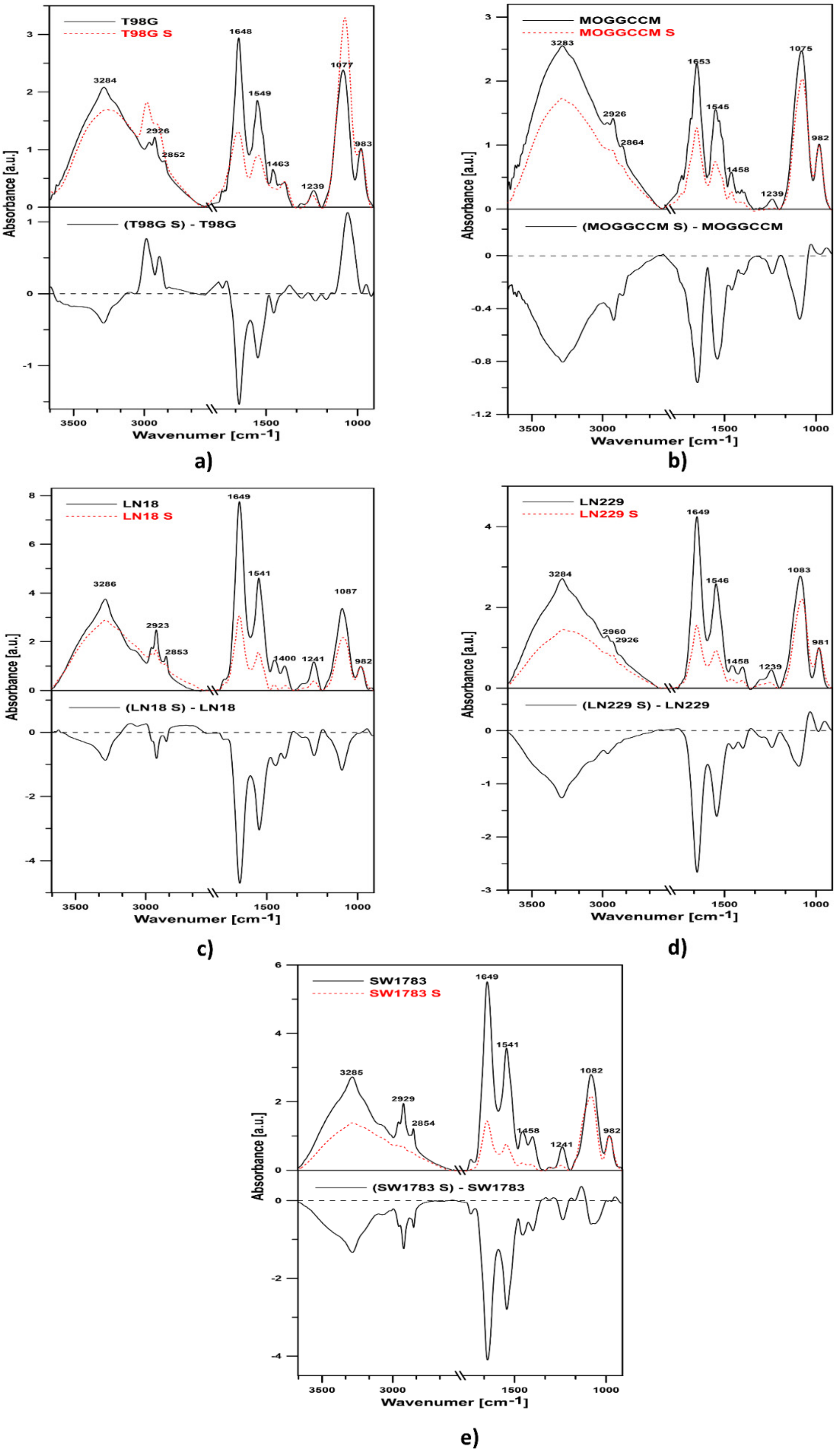

Figure S9. Efect of sorafenib applied alone in a) T98G, b) MOGGCCM, c) LN-18, d) LN229 and e) SW1783 cells. The top panels of graphs show spectra of control cells (black solid line) and cells treated with sorafenib (red, dashed line) for 24 hours. The lower panels present differential spectra of cells.

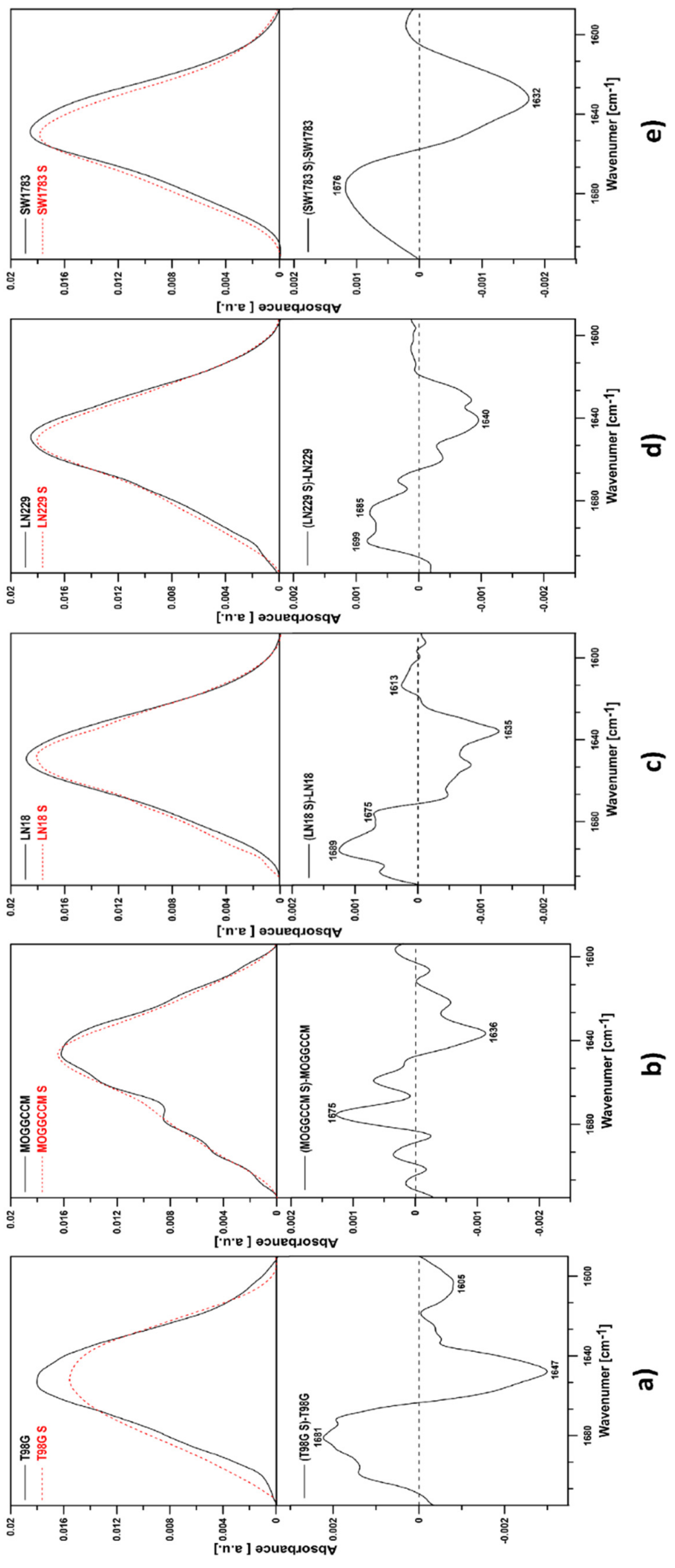

Figure S10. The amide I region (1700-1600 cm^{-1}) of control cells (upper panel, solid black line), cells incubated with sorafenib for 24 hours (upper panel, red, dashed line) and diffrential spectra (lower panel) from a) T98G, b) MOGGCCM, c) LN-18, d) LN229 and e) SW1783 line.
